# New Ophthalmosaurid Ichthyosaurs from the European Lower Cretaceous Demonstrate Extensive Ichthyosaur Survival across the Jurassic–Cretaceous Boundary

**DOI:** 10.1371/journal.pone.0029234

**Published:** 2012-01-03

**Authors:** Valentin Fischer, Michael W. Maisch, Darren Naish, Ralf Kosma, Jeff Liston, Ulrich Joger, Fritz J. Krüger, Judith Pardo Pérez, Jessica Tainsh, Robert M. Appleby

**Affiliations:** 1 Geology department, University of Liège, Liège, Belgium; 2 Paleontology Department, Royal Belgian Institute of Natural Sciences, Brussels, Belgium; 3 School of Earth and Environmental Sciences, University of Portsmouth, Portsmouth, United Kingdom; 4 School of Ocean and Earth Sciences, University of Southampton, Southampton, United Kingdom; 5 Staatliches Naturhistorisches Museum, Braunschweig, Germany; 6 Division of Environmental and Evolutionary Biology, School of Life Sciences, College of Medical Veterinary and Life Sciences, University of Glasgow, Glasgow, Scotland; 7 Institut für Geowissenschaften, Ruprecht-Karls-Universität Heidelberg, Heidelberg, Germany; University of Western Ontario, Canada

## Abstract

**Background:**

Ichthyosauria is a diverse clade of marine amniotes that spanned most of the Mesozoic. Until recently, most authors interpreted the fossil record as showing that three major extinction events affected this group during its history: one during the latest Triassic, one at the Jurassic–Cretaceous boundary (JCB), and one (resulting in total extinction) at the Cenomanian-Turonian boundary. The JCB was believed to eradicate most of the peculiar morphotypes found in the Late Jurassic, in favor of apparently less specialized forms in the Cretaceous. However, the record of ichthyosaurs from the Berriasian–Barremian interval is extremely limited, and the effects of the end-Jurassic extinction event on ichthyosaurs remains poorly understood.

**Methodology/Principal Findings:**

Based on new material from the Hauterivian of England and Germany and on abundant material from the Cambridge Greensand Formation, we name a new ophthalmosaurid, *Acamptonectes densus* gen. et sp. nov. This taxon shares numerous features with *Ophthalmosaurus*, a genus now restricted to the Callovian–Berriasian interval. Our phylogenetic analysis indicates that Ophthalmosauridae diverged early in its history into two markedly distinct clades, Ophthalmosaurinae and Platypterygiinae, both of which cross the JCB and persist to the late Albian at least. To evaluate the effect of the JCB extinction event on ichthyosaurs, we calculated cladogenesis, extinction, and survival rates for each stage of the Oxfordian–Barremian interval, under different scenarios. The extinction rate during the JCB never surpasses the background extinction rate for the Oxfordian–Barremian interval and the JCB records one of the highest survival rates of the interval.

**Conclusions/Significance:**

There is currently no evidence that ichthyosaurs were affected by the JCB extinction event, in contrast to many other marine groups. Ophthalmosaurid ichthyosaurs remained diverse from their rapid radiation in the Middle Jurassic to their total extinction at the beginning of the Late Cretaceous.

## Introduction

The thunnosaurian ichthyosaur *Ophthalmosaurus* Seeley 1874 [1] (family Ophthalmosauridae) is known from abundant material, most of it from the Oxford and Kimmeridge Clay formations of England and the Sundance Formation of the USA [2]. The widely accepted stratigraphic range for this taxon is Callovian-Tithonian [3], [4]. However, the presence of *Ophthalmosaurus* in Lower Cretaceous sediments has been claimed twice in the modern literature: McGowan [5] figured and discussed a humerus with three large distal facets from the Lower Cretaceous of Prince Patrick Island (Canada) that he referred to *Ophthalmosaurus* sp. and McGowan & Motani [2] mentioned the presence of isolated basioccipitals and humeri referable to *Ophthalmosaurus* in the early Cenomanian Cambridge Greensand Formation (which also includes a reworked late Albian fauna from the top of the Gault formation [6], [7]). These claims are, however, based on isolated material, and other ophthalmosaurid ichthyosaurs with three large distal humeral facets have been described from Cretaceous sediments since then, including *Caypullisaurus* [8], [9] and *Maiaspondylus* [10]. Therefore, the presence of *Ophthalmosaurus* in the Cretaceous remains ambiguous at best. This has important consequences for the evolution and diversity of Early Cretaceous ophthalmosaurids. Indeed, until recently [11], all Middle and Late Jurassic ichthyosaurs were thought to have become extinct at the end of the Jurassic, at the Jurassic–Cretaceous boundary [12]–[16], or during a more protracted extinction event that started during the Middle Jurassic [17]. The JCB, which is associated with climate change [18], [19], was therefore considered a major extinction event for ichthyosaurs, during which the successful “*ophthalmosaurs*” became extinct and replaced by what seemed to be less specialized forms. As summarized by Bakker [12]:

“*The Jurassic-Cretaceous boundary extinction disrupted ichthyosaur history profoundly – the hyper-specialized ophthalmosaur clade disappears, and the only Early Cretaceous ichthyosaurs, the platypterygians, are much more generalized, with longer bodies, smaller eyes, larger teeth and heavier snouts.*”

This contributed to the generally accepted idea that, despite their longevity (Olenekian, Early Triassic–Cenomanian, Late Cretaceous [20], [21]), ichthyosaurs underwent at least three major extinctions events throughout their history: during the Triassic–Jurassic boundary event [22], at the Jurassic–Cretaceous boundary (JCB), and during the Cenomanian–Turonian boundary event [20]. However, the worldwide record of ichthyosaurs from the Berriasian–Barremian interval is extremely limited, making preservation biases an important parameter to consider when analyzing the Jurassic–Cretaceous extinction event [11]. Yet, recent papers have highlighted the presence of some Late Jurassic ichthyosaurs in the Lower Cretaceous strata of South America, Europe, and Russia (*Caypullisaurus* [8], [9], *Aegirosaurus* [11] and the doubtful *Yasykovia* [23], respectively). The effect of the JCB extinction event on ichthyosaurs therefore remains poorly understood, whereas this extinction substantially affected several other groups related to the marine realm such as radiolarians, ammonites, marine crocodyliforms, pterosaurs, and plesiosaurs [12], [19], [24]–[28].

In order to examine the effect of the JCB extinction event on ichthyosaurs in detail and better understand the diversity and relationships of Early Cretaceous ophthalmosaurids, we:

1. Re-evaluate the presence of *Ophthalmosaurus* in the Cretaceous of Europe
2. Name a new Cretaceous ophthalmosaurid, *Acamptonectes densus* gen. et sp. nov., based on three well preserved specimens for a poorly sampled stage of the Early Cretaceous: the Hauterivian. This genus is also present in the Cambridge Greensand Formation (late Albian–early Cenomanian).
3. Propose a robust phylogenetic hypothesis for the evolution of ophthalmosaurids, which diverged early in its history into two clades: Ophthalmosaurinae and Platypterygiinae. Both clades crossed the JCB and persisted to the late Albian at least
4. Calculate cladogenesis, extinction, and survival rates for the Oxfordian–Aptian interval and show that the JCB event had a negligible impact on ichthyosaurs

## Materials and Methods

### Institutional abbreviations

CAMSM: Sedgwick Museum of Earth Sciences, Cambridge University, Cambridge, UK; CM: Carnegie Museum, of Natural History, Pittsburgh, PA, USA; GLAHM: The Hunterian Museum, University of Glasgow, Glasgow, UK; LEICT: New Walk Museum & Art Gallery, Leicester, UK; MHNH: Muséum d'Histoire naturelle du Havre, Le Havre, France; NHMUK: Natural History Museum, London, UK; OUM: Oxford University Museum; SCARB: Scarborough Trust Museums, Scarborough, UK; SNHM: Staatliches Naturhistorisches Museum Braunschweig, Braunschweig, Germany.

### Research history

#### The Speeton Clay specimens (GLAHM 132588, holotype of A. densus; NHMUK R11185, one paratype of A. densus)

The specimen GLAHM 132588, mainly known as “the Speeton Clay ichthyosaur”, has a long research history filled with gaps. The specimen was collected over two weekends in Spring 1958 for the Geology Department of Hull University by a group of four final year geology undergraduates: J Keith Ingham, Cyril Haskins, John Wilkins, Mike Golding with departmental technician Pete Robinson. Neale [29] mentioned it in 1968. RMA had skull elements (and some sample centra) on loan prior to 1989. When the Geology Department was closed as part of the Earth Sciences Review in 1991, the specimen was transferred to the Hunterian Museum of the University of Glasgow, where J Keith Ingham was then employed. RMA described the specimen with great detail and referred it to “*Platypterygius speetoni*” in his monograph, which remains unpublished because of its death in 2003. RMA considered GLAHM 132588 as a particularly primitive species of *Platypterygius*. The second Speeton Clay specimen (NHMUK R11185) was found in 1985 the locality of Filey, 6 miles north of Speeton.

#### The Cremlingen specimen (SNHM1284-R, one paratype of A. densus)

The private collector Hans-Dieter Macht discovered the Cremlingen specimen during collecting fossils at the construction area of a new autobahn (A39) in the vicinity of Cremlingen, Northern Germany, in May 2005. After finding some isolated vertebrae the collector informed the director of the SNHM in Braunschweig and the excavation began immediately. Since the constructional works had to go on the crew was forced to finish the excavation within only three days of fieldwork. Afterwards, FJK and RK prepared and mounted the specimen for a special exhibition at the SNHM in fall 2005. Since 2006, the Cremlingen specimen is housed at the paleontological collection of the SNHM.

### Geological context

The specimen known as the “Speeton Clay ichthyosaur” (GLAHM 132588) originates from the ‘D2D beds, *Acroteuthis subquadratus* and *Hibolites jaculoides* beds’ of the Berriasian to Albian Speeton Clay Formation [29]. In the Speeton area, this horizon reworks material from the upper Valanginian [30], but since the ichthyosaur was found partially articulated and in association with nearly complete crinoids [29], it must come from the unreworked part of the D2D beds, which is basal Hauterivian in age [30], [31]. A second Speeton Clay ichthyosaur is present in the collections of NHMUK (NHMUK R11185) and originates from the D2C bed. It is therefore slightly older than GLAHM 132588, but still early Hauterivian [30], [32]. Marine reptile remains are rare in the Speeton Clay Formation: GLAHM 132588 and NHMUK R11185 are the first ichthyosaurs reported from this formation. However, at least three fragmentary plesiosaurs have also been unearthed from the Hauterivian beds of this formation (SCARB 2007.51, NHMUK R6650, and NHMUK 48623; [Forrest, pers. com. June 2011]). Generally, the record of marine reptiles from the Berriasian–Barremian interval is highly limited, especially concerning ichthyosaurs. The Cremlingen specimen (SNHM1284-R) is late Hauterivian in age. See Seibertz & Krüger [33] for precise stratigraphic data on this specimen.

### Phylogeny

We modified the phylogenetic matrix compiled by Fischer et al. [34]. We deleted two dental characters (chars. 4 and 5) because examination of the abundant material of *Ophthalmosaurus* showed that these characters varied during ontogeny. We also deleted character 23 because the states were not clearly defined and proved uninformative. Characters 3 and 34 were slightly modified (see Text S1) as were some codings: this is due both to new observations, and because the analysis now incorporates species-level taxa amongst Thunnosauria (character-taxon matrix available in Table S1 and nexus file in Text S2). Finally, we added four new characters (chars. 19, 20, 27, 36, see Text S1) and two taxa: *Ophthalmosaurus natans* and *Acamptonectes densus*. Characters were coded from the literature and personal observations for *Temnodontosaurus* (IRSNB R122 and IRSNB R123), *Platypterygius hercynicus* (MHNH 2010.4 and a cast of the holotype held at the SNHM), *Sveltonectes insolitus* (IRSNB R269), *Ophthalmosaurus natans* (CM material), *Ophthalmosaurus icenicus* (NHMUK and GLAHM material) and *Acamptonectes densus* (GLAHM 132588, SNHM1284-R, NHMUK R11185). We used exact parsimony searches of TNT v1.1 [35] to analyze the character matrix (see supporting information) and calculate the Bremer support and bootstrap values. We generated the phylogenetic tree with unambiguous optimization using Winclada v.0.9 [36] (fast and slow optimizations are available in Figure S1). Characters were not weighted and, except for characters 17, 39, and 45, were not ordered.

### Calculating cladogenesis, extinction, and survival rates

We counted the number of clades that appear and genera that disappear at each stage boundary for the Oxfordian–Barremian interval, and assumed that each taxon appeared or disappeared at one of these boundaries. For example, the current stratigraphic range of *Caypullisaurus bonapartei* is Tithonian–Berriasian; we therefore incremented the cladogenesis count of the Kimmeridgian–Tithonian boundary (because *C. bonapartei* is the oldest representative of a clade grouping *C. bonapartei*, *P. australis* and *A. bitumineus* according to our phylogenetic analysis) and incremented the extinction count of the Berriasian–Valanginian boundary. We used a ‘per boundary count’ instead of the usual ‘per interval count’ because we feel it seems more logical considering recent advances in the extinction theories such as the ‘common cause’ hypothesis [37], [38]. Fossil occurrences and Lazarus taxa are considered for each time bin and for each currently valid genus (i.e. those used in the phylogenetic analysis + *Nannopterygius*). This simple count of cladogenesis/survival/extinction rates at the stage level is not biased by differential time bins since the stages of the Oxfordian–Barremian interval have roughly the same duration (mean = 5.17 Ma, standard deviation = 0.77). We calculated ‘survival rates’ by counting the genera and lineages that cross each stage boundary.

We considered two scenarios: a conservative one in which the post-Jurassic remains of both *Ophthalmosaurus* (the Nettleton material, see below) and *Brachypterygius* (OUM J.13795 from the Berriasian of England [39], and *Brachypterygius cantabrigiensis* from the Albian of England) were ignored, and a ‘total evidence’ one in which these remains were considered valid occurrences of these genera. Data were not corrected with respect to the quantity of specimens or of marine geological formations, because we wanted to show that even the raw data, which include an obvious bias in the earliest Cretaceous record, are sufficient to challenge the existence of an extinction event at the JCB for ichthyosaurs.

### Nomenclatural Acts

The electronic version of this document does not represent a published work according to the International Code of Zoological Nomenclature (ICZN), and hence the nomenclatural acts contained in the electronic version are not available under that Code from the electronic edition. Therefore, a separate edition of this document was produced by a method that assures numerous identical and durable copies, and those copies were simultaneously obtainable (from the publication date noted on the first page of this article) for the purpose of providing a public and permanent scientific record, in accordance with Article 8.1 of the Code. The separate print-only edition is available on request from PLoS by sending a request to PLoS ONE, Public Library of Science, 1160 Battery Street, Suite 100, San Francisco, CA 94111, USA along with a check for $10 (to cover printing and postage) payable to “Public Library of Science”.

An electronic version of this document is deposited in the institutional online repository of the University of Liege: ORBi (http://orbi.ulg.ac.be).

## Results

### Systematic Paleontology

Ichthyosauria Blainville 1835

Neoichthyosauria Sander 2000

Thunnosauria Motani 1999

Ophthalmosauridae Baur 1887

*Acamptonectes* gen. nov.

urn: lsid:zoobank.org:act:87EA8F31-7752-4968-970A-A43A3C71D5E4

*Acamptonectes densus* sp. nov.

urn: lsid:zoobank.org:act:AAB4BA7E-F53D-4962-8A9C-3240DFE2C4D8

**Figure 1:**
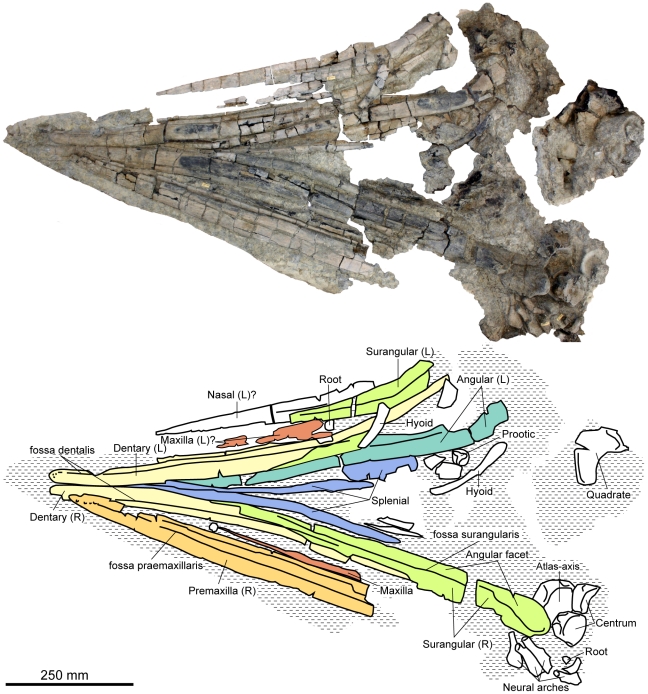
Skull and partial cervical region of *Acamptonectes densus* (SNHM1284-R).

Figures 1–10

**Figure 2 pone-0029234-g002:**
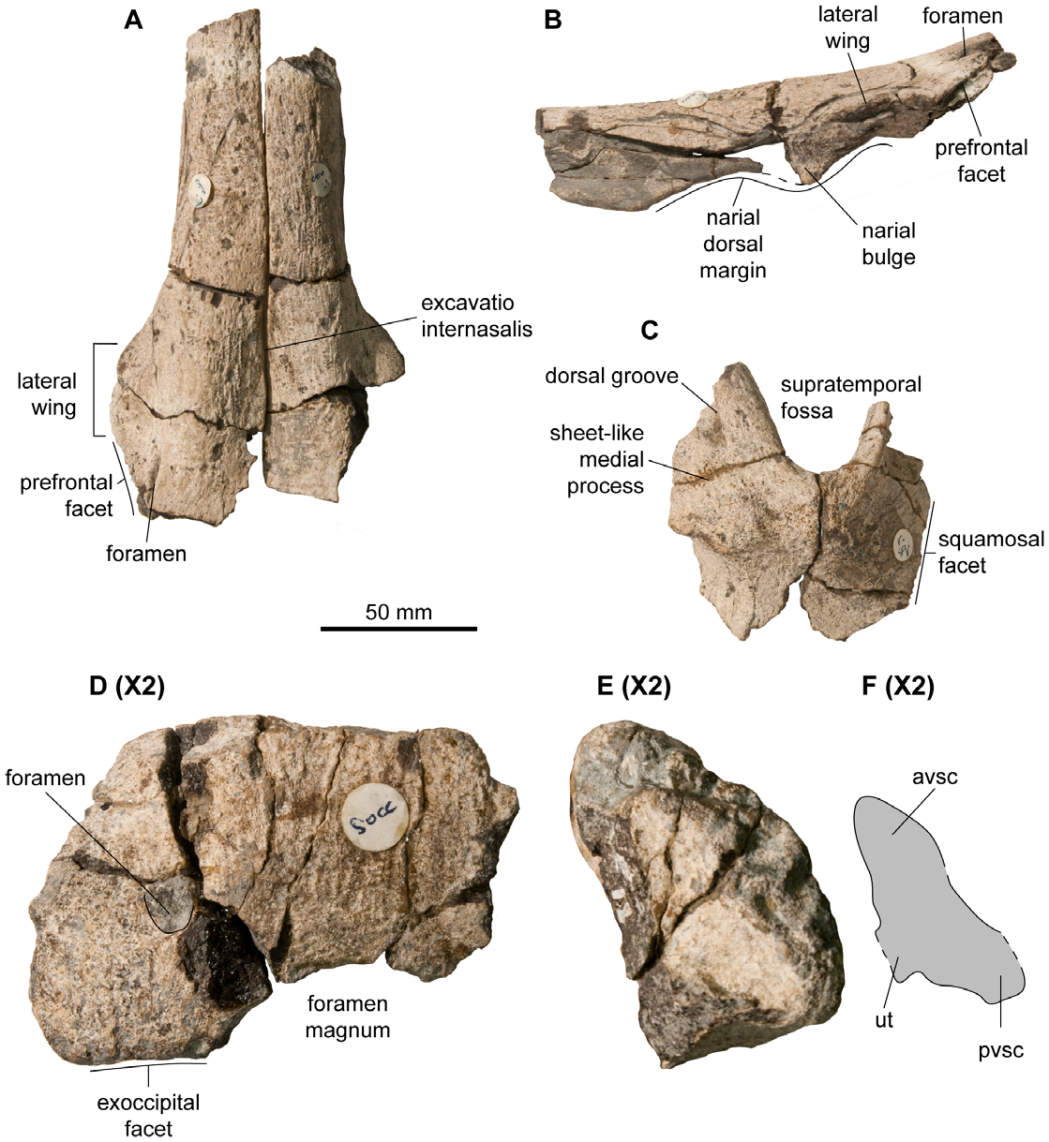
Skull roof of *Acamptonectes densus* (GLAHM 132588, holotype). A: articulated nasals in dorsal view. B: left nasal in lateral view. C: right supratemporal in dorsal view. D–F: supraoccipital magnified two times with respect to the other bones, in posterior view (D) and in left anterolateral (otic) view (E,F). Note the lateral wing of the nasal forming an overhang on the naris, the narial process of the nasal, the long and straight squamosal facet of the supratemporal, and the weakly arched shape of the supraoccipital. Abbreviations: avsc: impression of the anterior vertical semicircular canal; pvsc: impression of the posterior vertical semicircular canal; ut: utriculus.

**Figure 3 pone-0029234-g003:**
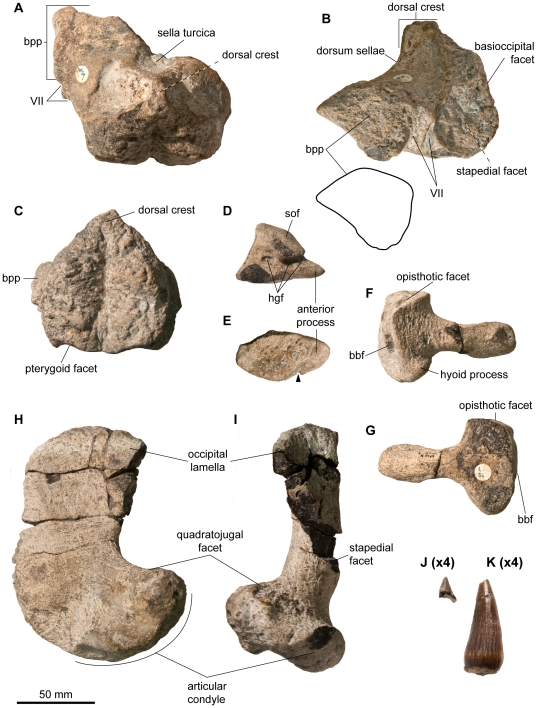
Basicranium, quadrate, and dentition of *Acamptonectes densus* (GLAHM 132588, holotype). A–C: basisphenoid, in dorsal view (A), in lateral view (B), and in posterior view (C). The thick and claw-like shape of the basipterygoid process in lateral view in shown in B. Note the dorsal crest and the paired facialis (VII) nerve foramen. D,E: left exoccipital, in medial view (D) and ventral view (E). The arrow indicates a notch that matches a small bump on the dorsal surface of the basioccipital, suggesting a close fit of these bones (and therefore a thin cartilage layer) that we interpret as indicator of a mature age. F,G: left stapes, in anteromedial view (F) and posterolateral view (G). Note the slenderness of the shaft compared to the occipital head. H,I: left quadrate, in lateral view (H) and posterior view (I). J,K: fragmentary tooth crowns magnified four times with respect to the other bones. Note the subtle striations and the constriction at the base of the crown in K. Abbreviations: bbf: facet for basioccipital and basisphenoid; bpp: basipterygoid process; hgf: hypoglossal foramina; sof: supraoccipital facet; VII: foramen for the facialis nerve (VII).

**Figure 4 pone-0029234-g004:**
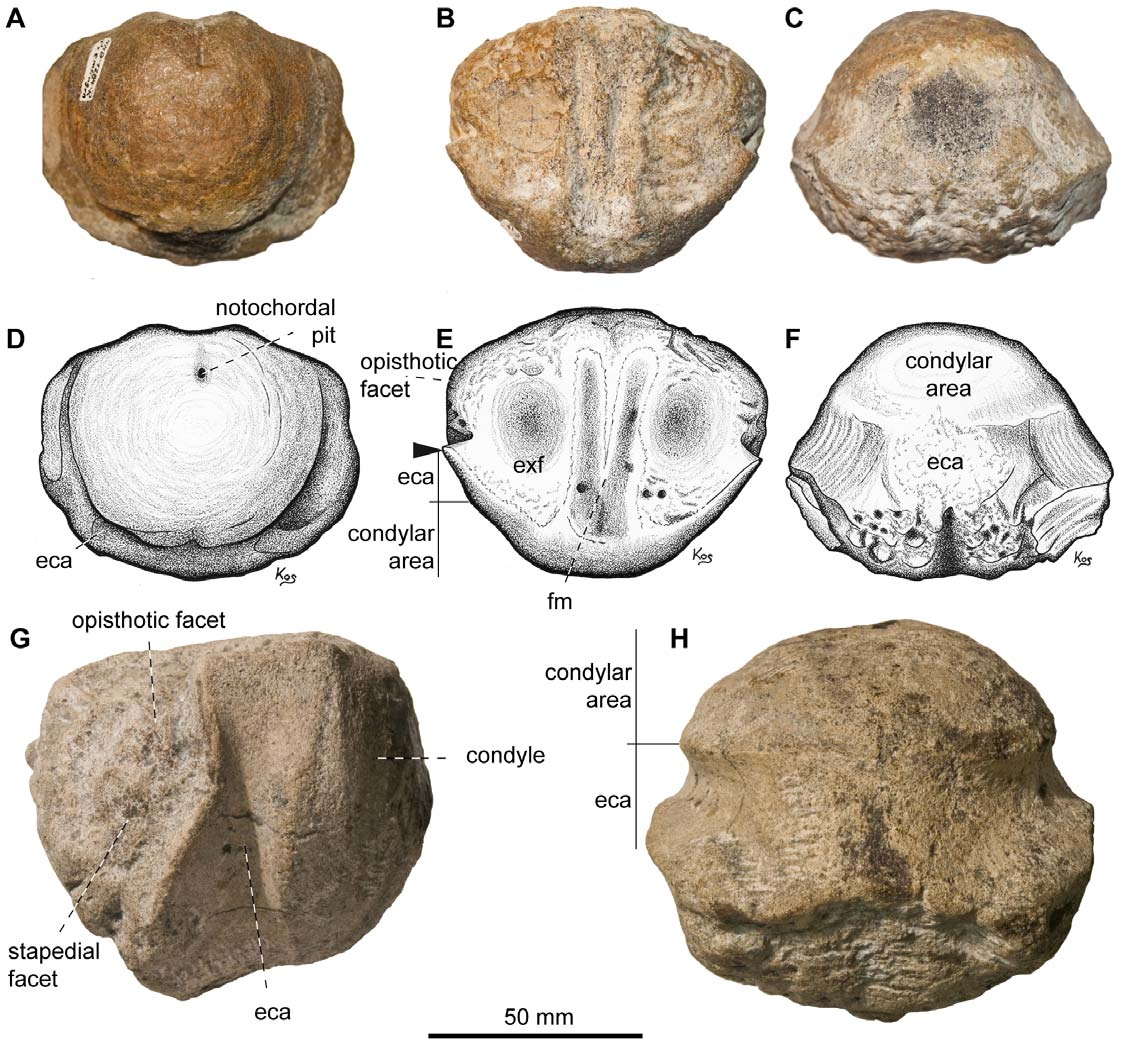
Basioccipital of *Acamptonectes densus*. A–F: SNHM1284-R, in posterior view (A,D), in dorsal view (B,E), and in ventral view (C,F). G–H: GLAHM132588 (holotype), in lateral view (G) and ventral view (H). Note the markedly concave extracondylar band that separates the condyle from the rest of the basioccipital and the bilobed median concavity for the foramen magnum. The arrow in E indicates the protruding anterior edge of the extracondylar area posterior to the depressed opisthotic facet. Abbreviations: eca: extracondylar area; exf: exoccipital facet; fm: median concavity for the foramen magnum.

**Figure 5 pone-0029234-g005:**
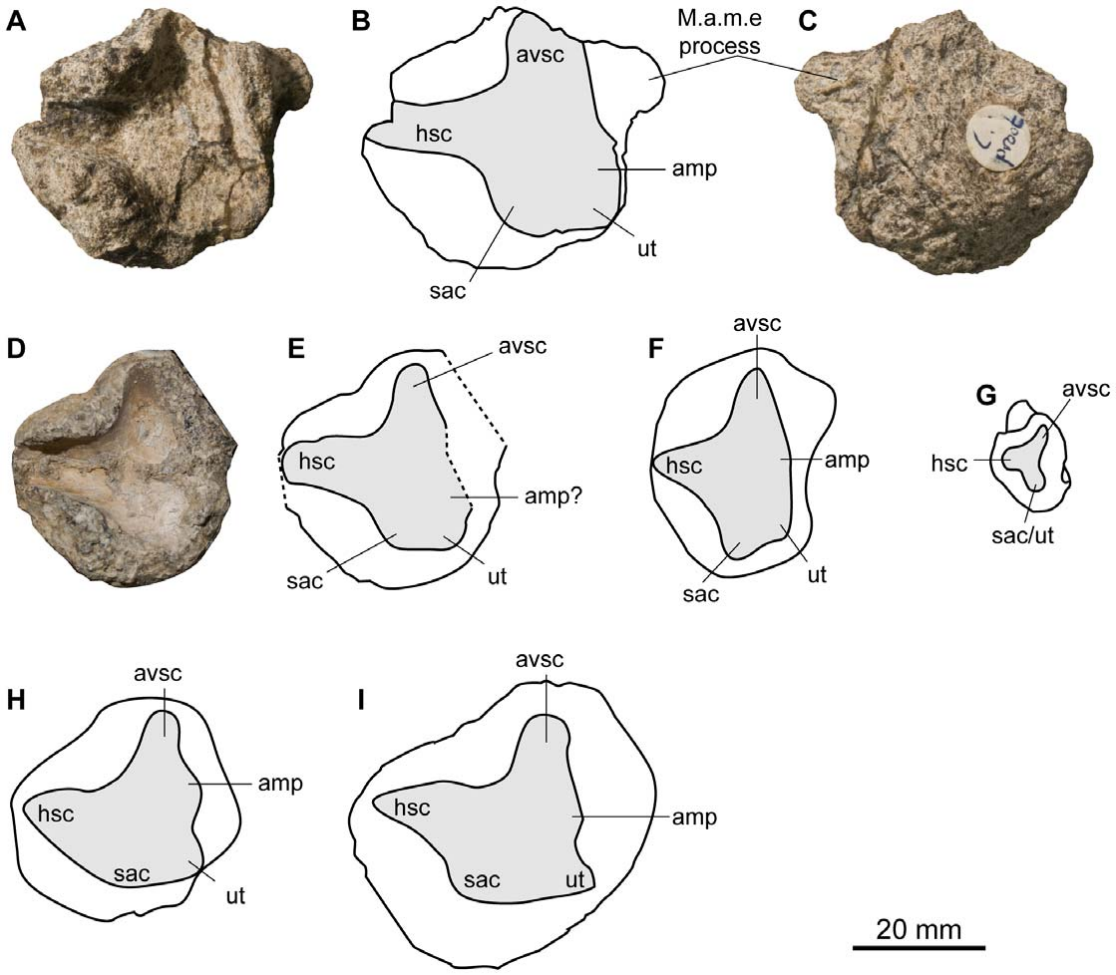
Left prootic of *Acamptonectes densus* compared to that of other ophthalmosaurids. A–C: *A. densus* (GLAHM 132588, holotype), in posterior view (A,B) and anterior view (C). D,E: *A. densus* (SNHM1284-R), in posterior view. F: *Platypterygius australis* (QMF14339), in posterior view redrawn from Kear [58]. G: *Sveltonectes insolitus* (IRSNB R269, holotype), in posterior view, from Fischer et al. [34]. H: *Ophthalmosaurus icenicus* (NHMUK R4522, mirrored), in posterior view, redrawn from Kirton [43]. I: *Ophthalmosaurus icenicus* (NHMUK R2161), in posterior view, redrawn from Andrews [51]. Abbreviations: amp: ampulla; avsc: impression of the anterior vertical semicircular canal; hsc: impression of the horizontal semicircular canal; M.a.m.e. facet: facet for attachment of musculus adductor mandibulae externus; sac: sacculus; ut: utriculus.

**Figure 6 pone-0029234-g006:**
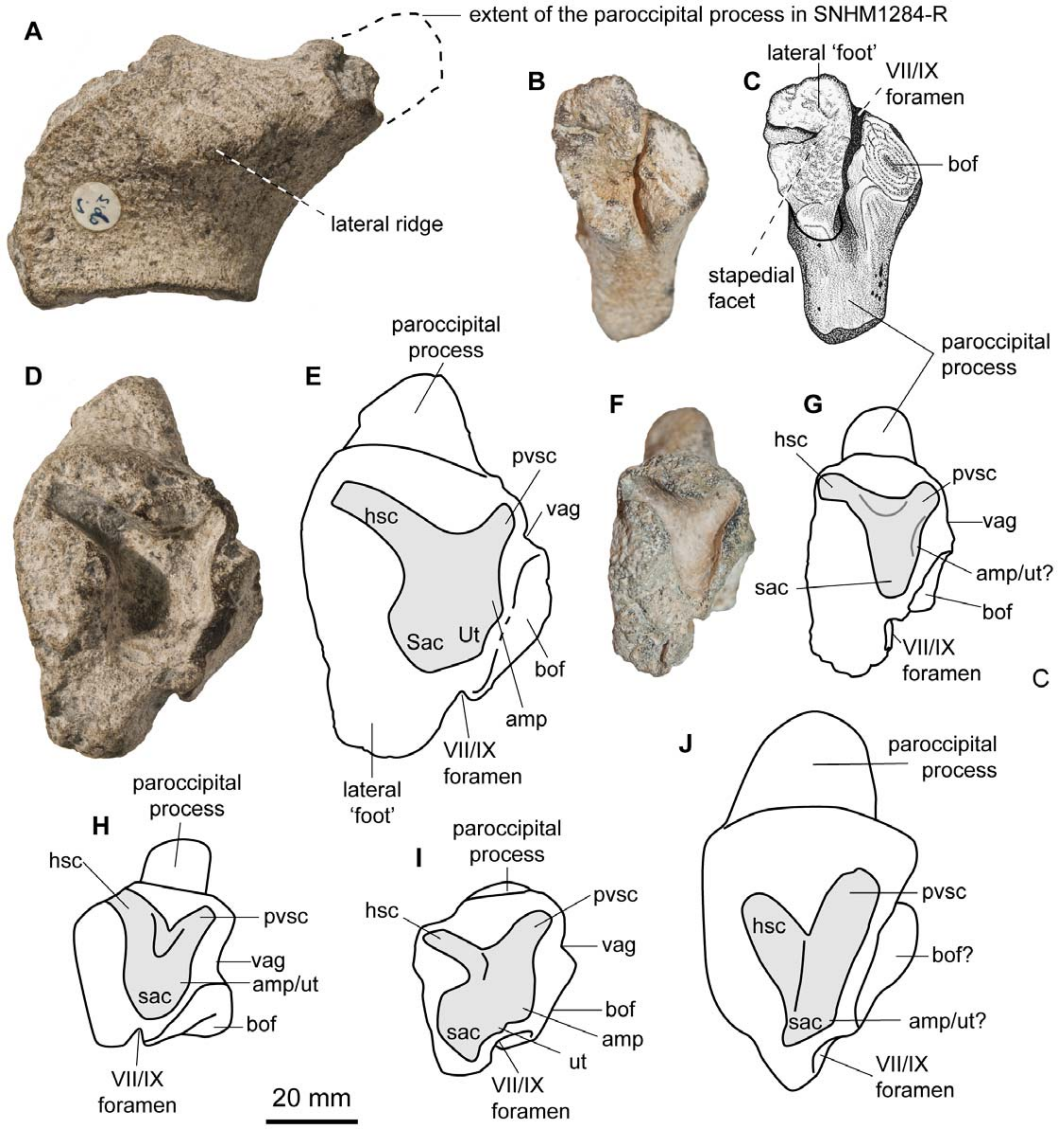
Right opisthotics of *Acamptonectes densus* compared to that of other ophthalmosaurids. A: *A. densus* (GLAHM 132588, holotype), in posterolateral view showing the lateral ridge. B,C: *A. densus* (SNHM1284-R), in ventral view showing the peculiar thickened lateral foot. D,E: *A. densus* (GLAHM 132588, holotype), in otic (anteromedial) view. F,G: *A. densus* (SNHM1284-R), in otic view. H: *Ophthalmosaurus icenicus* (NHMUK R4523), in otic view, redrawn from Kirton [43]. I: *Platypterygius australis* (AM F98273), in otic view, redrawn from Kear [58]. J: *Mollesaurus periallus* (MOZ 2282, holotype), in otic view, redrawn from Fernández [45]. Abbreviations: amp: ampulla; bof: basioccipital facet; hsc: impression of the horizontal semicircular canal; pvsc: impression of the posterior vertical semicircular canal; sac: sacculus; ut: utriculus; vag: vagus foramen.

**Figure 7 pone-0029234-g007:**
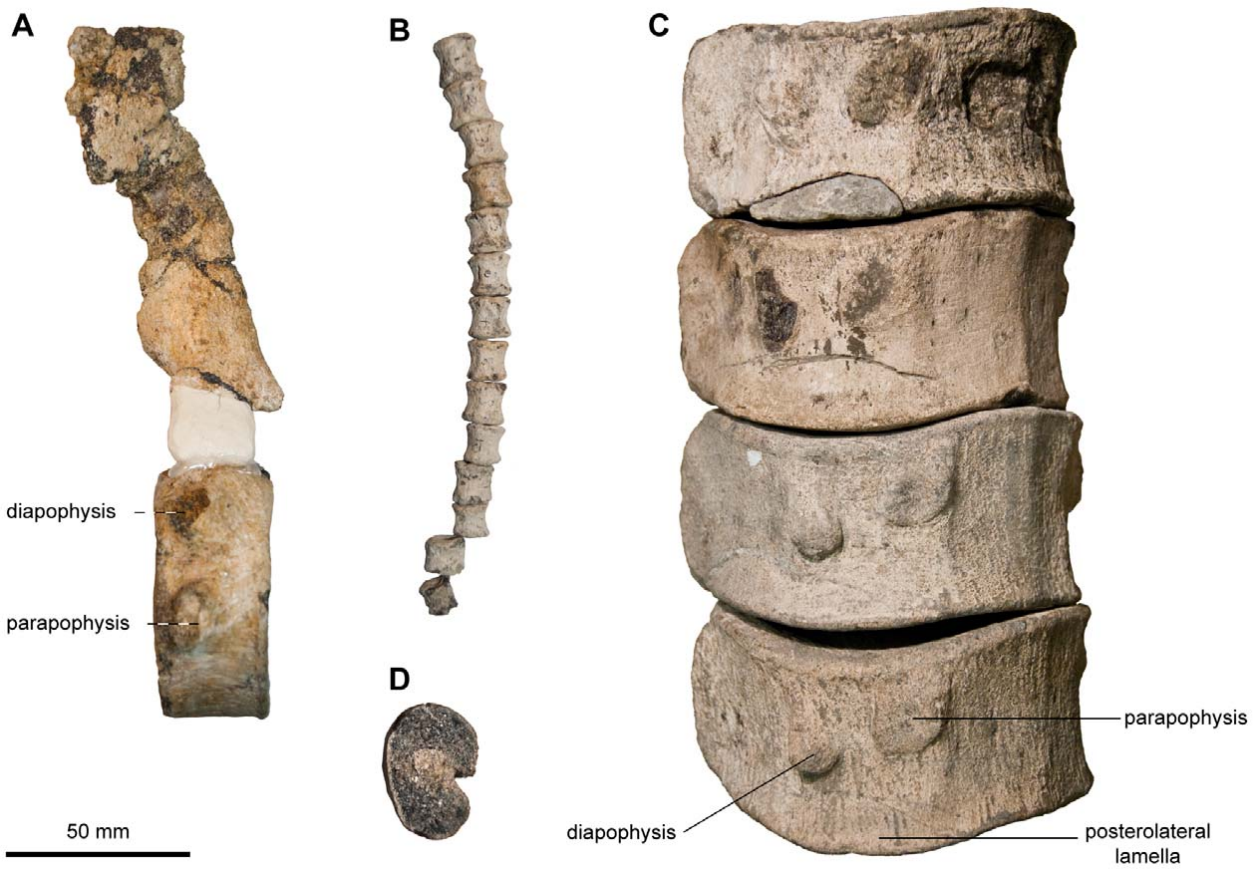
Diagnostic features of the axial skeleton of *Acamptonectes densus*. A: anterior dorsal centrum and associated neural spine of SNHM1284-R, showing the large size of the neural spine with respect to the centrum height. B: series of posterior postflexural centra of SNHM1284-R showing their square shape (H/L≈1). C: series of posterior dorsal centra of GLAHM 132588 (holotype), showing the markedly curved profile of the posterolateral lamella. D: cross-section of a rib of NHMUK R11185 showing their robust morphology and the minute groove occurring on one side only.

**Figure 8 pone-0029234-g008:**
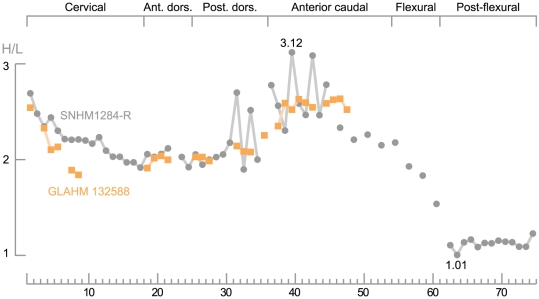
Regionalization of the vertebral column in *Acamptonectes densus*. Both specimens have incomplete vertebral series and the centra were therefore superposed manually in each region, using the centrum shape and relative position of the apophyses. Each obvious gap in the vertebral column is represented by a single void in the series of SNHM1284-R. The x-axis refers to the centrum count, not its actual position within the vertebral column. The regionalization of the vertebral column is quite weak, in between that of *Sveltonectes insolitus* [34] and *Ophthalmosaurus icenicus* [69].

**Figure 9 pone-0029234-g009:**
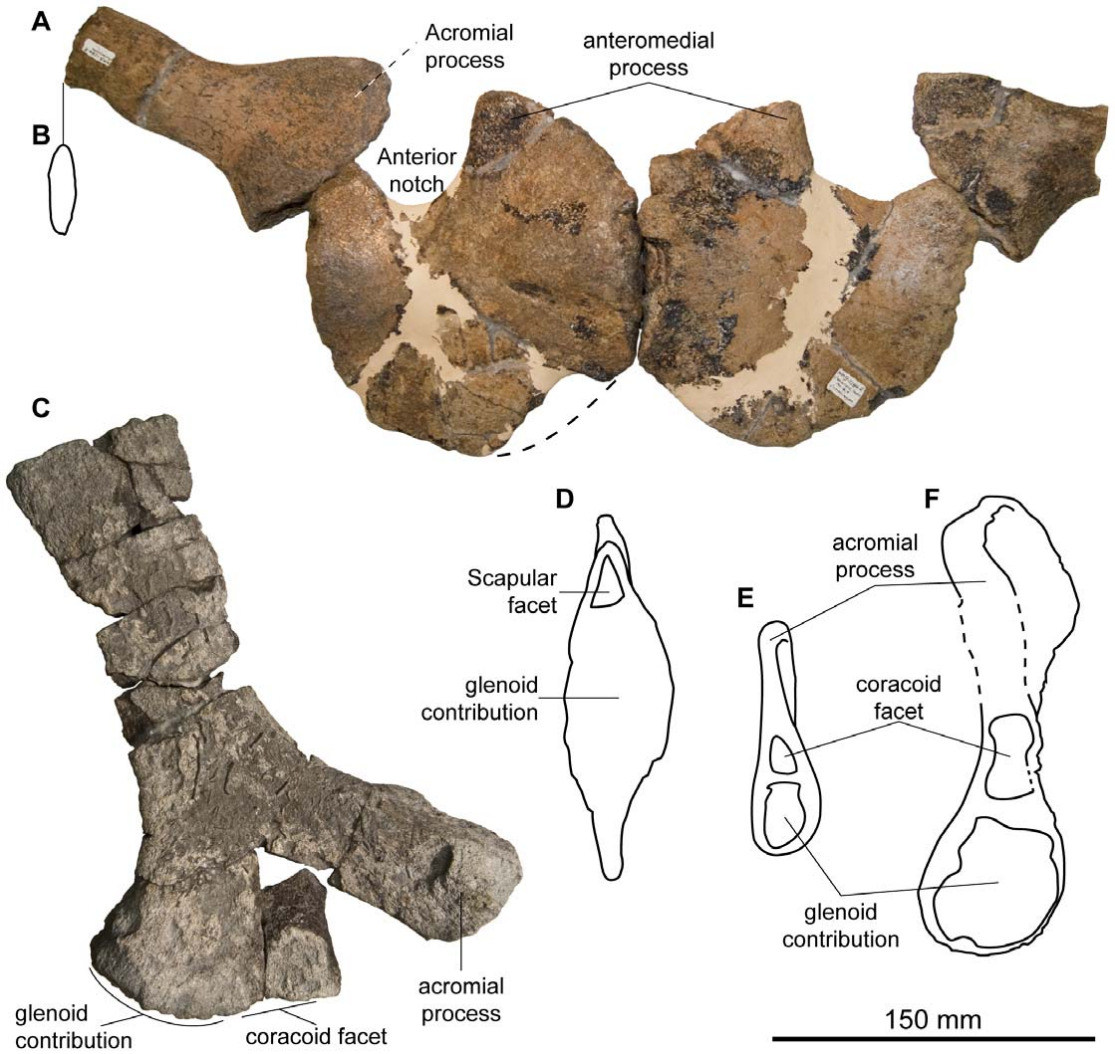
Scapular girdle of *Acamptonectes densus*. A: coracoids and scapulae of SNHM1284-R in ventral view. B: Outline of the cross-section of the scapula, showing its flattened shape. C: right scapula of GLAHM 132588 (holotype), in ventral view. D: lateral surface of the right coracoid of SNHM1284-R. E,F: comparison of the medial surface of the right scapula of SNMM1284-R (E) and of GLAHM 132588 (holotype; F).

**Figure 10:**
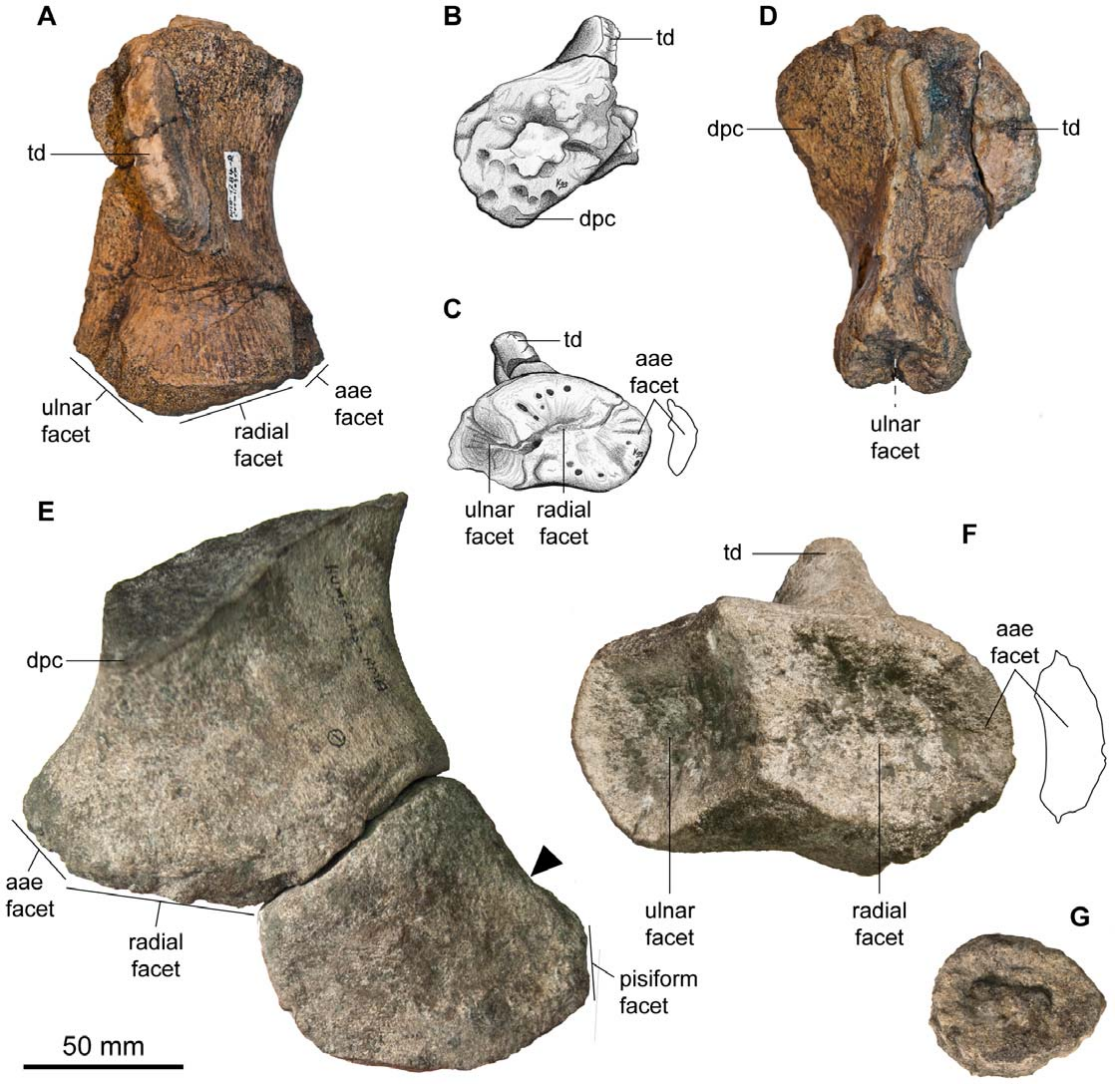
Forefin of *Acamptonectes densus*. A–D: right humerus of NBM1284-R, in dorsal view (A), proximal view (B), distal view (C), and posterior view (D). E,F: right humerus of GLAHM 132588 (holotype), in ventral view with the associated ulna (E) and in distal view (F). The arrow points at the concave and edge-like posterior surface of the ulna, diagnostic of ophthalmosaurine ophthalmosaurs. G: proximal phalange of GLAHM 132588 (holotype). Abbreviations: aae facet: facet for the anterior accessory element; dpc: deltopectoral crest; td: trochanter dorsalis.

#### Etymology

From Greek ‘akamptos’ and ‘nektes’, which means rigid swimmer and from Latin ‘densus’, wich means compact, tightly packed. This binomial refers to the robust and tightly fitting bones of the occiput and to the tightly interlocking cervical and dorsal centra.

#### Diagnosis

Ophthalmosaurid characterized by the following autapomorphies: bilobed median concavity of the basioccipital for the foramen magnum; stapes with slender, rod-like shaft and large cubic occipital head; prominent, crest-like dorsal surface of basisphenoid; tightly interlocking dorsal centra with extensive posterolateral lamella; elongate thoracic neural spine >1.3 times the height of corresponding centra; ribs rounded in cross-section with a single deep proximal groove.

*Acamptonectes densus* is also characterized by the following unique combination of features: presence of a narial process on the nasal (shared with *Ophthalmosaurus* [40], [41], *Aegirosaurus* [42], and *Sveltonectes* [34]); slender paroccipital process (shared with *Ophthalmosaurus icenicus* [43]); V-shaped otic capsule impression on the opisthotic (shared with *Ophthalmosaurus* [41], [43], [44] and *Mollesaurus* [45]); condyle demarcated from the body of the basioccipital by a concave extracondylar area (shared with *Ophthalmosaurus* [41], [43], [44] and *Mollesaurus* [45]); triangular exoccipital with expanded occipital foot (shared with *Brachypterygius* [46], *O. icenicus* [43] and *Mollesaurus* [45]); high number of ‘cervical’ centra (shared with *Platypterygius americanus* [47], ‘*Otschevia*’ [48] and *Sveltonectes* [34]); postflexural centra as high as long (shared with *Platypterygius platydactylus* [49]); hexagonal coracoid with an anteromedial process and a wide anterior notch (shared with *Ophthalmosaurus* [41], [44], [50]); scapula with fan-shaped acromial process (shared with *Ophthalmosaurus* [43], [51] and *P. americanus* [47]); high and narrow trochanter dorsalis (shared with *Sveltonectes* [34] and many species of *Platypterygius* [52]–[54]); humerus with three distal facets, including a facet for an anterior accessory element and a posteriorly deflected ulnar facet (shared with *Ophthalmosaurus* [41], [43], [50] and *Arthropterygius* [55]); ulna with concave and edge-like posterior margin (shared with *Ophthalmosaurus* spp. [41], [43]), oval phalanges (shared with *Ophthalmosaurus* spp. [41], [51], *Arthropterygius* [55] and some specimens of *Brachypterygius* [43]).

#### Holotype

GLAHM 132588, a partial adult skeleton, including fragmentary skull roof, mandible, axial skeleton and scapular girdle.

#### Paratypes

SNHM1284-R, a partial subadult skeleton, including fragmentary skull roof, complete mandible, partial axial skeleton and partial scapular girdle; NHMUK R11185, a partial rostrum and mandible, fragmentary ribs and a complete right humerus.

#### Stratigraphic range

D2D horizon of the Speeton Clay Formation, basal Hauterivian – *Simbiskites concinnus*/*staffi* zone, upper Hauterivian, Early Cretaceous.

#### Geographical range

Speeton and Filey area, North Yorkshire, UK – Cremlingen area, Lower Saxony, Germany.

### Description

SNHM1284-R is a subcomplete ophthalmosaurid comprising a crushed skull and mandible, an incomplete axial skeleton, and a partial scapular girdle. The proximal surface of the capitulum of the humerus is deeply pitted and slightly flattened peripherally, which suggests immaturity [56]. However, the shaft of the humerus lacks the sandpaper-like texture present on the bones of juvenile ichthyosaurs [ibid.], and the specimen is therefore considered to be a subadult. GLAHM 132855 is a large incomplete ophthalmosaurid lacking most of the skull roof and the posterior half of the skeleton. It is a fully ossified adult, as suggested by the closely fitting bones of the occiput (exoccipital–basioccipital, parasphenoid-basisphenoid, basisphenoid–pterygoid, and opisthotic–stapes) and the smooth texture of the humerus. NHMUK R11185 is a large incomplete ophthalmosaurid, comprising a right humerus, fragmentary rostrum and mandible, and fragmentary ribs.

Measurements are provided in Table 1 and ratios in Table 2. Table 3 lists features that vary intraspecifically between the holotype (GLAHM 132588) and one of the paratypes (SNHM1284-R) and that have not been related to ontogeny before.

**Table 1:** Selected measurements of the holotype (GLAHM 132588) and of one paratype (SNHM1284-R) of *Acamptonectes densus*.

Measurement (mm)	SNHM1284-R	GLAHM 132855
Mandible length	1045	/
Premaxilla height at mid length	46.6	/
Quadrate height	133.8	142.1
Quadrate articular condyle width	50	65.1
Basioccipital width	77.05	91.6
Basioccipital height	57.9	73.8
Basioccipital length	61.95	84
Basioccipital condyle width	58	72
Exoccipital foot length	/	54.15
Exoccipital height	/	37.35
Highest neural spine	101.9	/
Coracoid length	174.6	/
Coracoid width	104	/
Coracoid depth	29.5	/
Scapula length	104	182.7
Radial facet length	35	48.7
Ulnar facet length	34.5	58
AAE facet length	9.5	15.5

Measurements up to 210 mm are recorded up to the nearest 0.05 mm using a plastic caliper. Measurements above 210 mm are recorded up to the nearest 1 mm using a meter.

**Table 2:** Ratios of taxonomic importance of the ophthalmosaurine ichthyosaurs discussed in the text.

Ratios	SNHM1284-R	GLAHM 132855	NHMUK R11185	CAMSM B57942	LEICT G1.2001.016
Snout depth ratio	0.0446	/	/	/	/
Extracondylar width ratio (%)	24.72	14.83	/	35.15	/
Length of AAE facet/length radial facet (%)	27.09	31.83	31.57	/	44.9

*Acamptonectes densus* (GLAHM 132588, SNHM1284-R, NHMUK R11185), *Acamptonectes* sp. (CAMSM B57942), and cf. *Ophthalmosaurus* (LEICT G1.2001.016).

**Table 3:** Variable morphological features in *Acamptonectes densus*.

Feature	State in SNHM1284-R (subadult)	State in GLAHM 132588 (adult)
Ossification of the paroccipital process of the opisthotic	Complete	Incomplete (cartilaginous end)
Impression of the sacculus on the opisthotic and the prootic	Weak	Marked
Hyoid process on the stapes	Absent	Variable
Prominence of the ventral rim of the extracondylar area of the basioccipital	Weak	Marked
Anterior surface of the basioccipital	Presence of shallow notochordal groove	No groove but numerous bulge-like processes
M.a.m.e. process on surangular	Absent	Present
‘3’-shaped dorsal surface of the angular	Absent	Present
Undulated profile of the thoracic centra	Weak	Marked
Shape of the acromial process of the scapula	Straight lamella	Undulating lamella
Deltopectoral crest of the humerus	Small, does not extend beyond mid-shaft	Tall, extends far beyond mid-shaft
Posterior surface of the humerus	Acute trailing blade	Acute but rounded
Largest distal facet on humerus	Radial facet	Ulnar facet

The features listed here are those not previously assigned to ontogeny: typical ontogenetic features (such as sandpaper-like texture on the humerus, the shape of the capitulum of the humerus, and the overall ‘fit’ of the basicranium and forefin bones) are discussed in the text. Potentially, the variations listed here could be interpreted as representing ontogenetic, phyletic, or intraspecific variation, given the differences in ontogenetic stage (subadult VS adult) and stratigraphic age (late Hauterivian VS basal Hauterivian) of the two specimens. Because we interpret both individuals as representatives of the same species, we propose that the morphological features listed here are within normal intraspecific variation and hence possibly of limited phylogenetic interest.

#### Skull roof

The premaxilla (SNHM1284-R, NHMUK R11185; Figure 1) is elongated and thin (snout depth ratio ca. 0.044, one of the lowest of all ophthalmosaurids: 0.047 in *Aegirosaurus* [11], 0.043 in *P. americanus* [46]). The fossa praemaxillaris is deep and continuous. Anteriorly, this groove ends as a series of aligned foramina. The dental groove exhibits slight demarcation into subtle alveoli anteriorly, as is also the case in *Aegirosaurus* [11], *Ophthalmosaurus* [3], and some *Platypterygius* species [57], [58]. Posteriorly, the ventral surface of the dentary groove's labial wall is flattened and slightly striated, indicating the attachment area for the missing maxilla. This flattened area appears extensive in NHMUK R11185.

The maxilla (GLAHM 132588, SNHM1284-R; Figure 1) is laterally compressed and, in cross-section, is similar to that of *O. icenicus* [51]: ventrolaterally, the maxilla is sheet-like and forms the internal part of the labial wall and the dorsal part of the deep dental groove. The maxilla is flat and smooth laterally, whereas it forms a prominent trapezoidal palatine process medially. Dorsal to this process, the maxilla forms a dorsal groove that separates the palatine plate from the dorsal narial ramus. The dorsal edge of the narial ramus is smooth and flat: this indicates that this fragment is located anterior to the narial aperture, since the narial ramus of the maxilla usually forms a prominent process [51], [58]. On the right side, a maxillary facet is present on the labial wall of the dental groove of the premaxilla, but its anterior extent, which is sometimes used as a diagnostic feature [2], [59], is unknown.

The nasals (GLAHM 132588; Figure 2A, 2B) are three-dimensionally preserved and only weakly deformed, which allows to reconstruct the shape of the dorsal half of the rostrum. The snout is extremely slender, being only 45 mm wide just anterior to the level of the naris. The flat internasal surface is thickest just before the beginning of the excavatio internasalis. The dorsal margin of the naris is slightly concave anteriorly. Posteriorly, the nasal forms a ventral bulge like that present in *Aegirosaurus* [42], *Ophthalmosaurus* spp. [41], [51] and *Sveltonectes* [34]. A thick, short lateral wing originates from this bulge. This wing forms an overhang on the posterodorsal part of the external naris, as it also does in *O. icenicus* [51] and *P. australis* [58]. The ventral surface of this lateral wing bears a roughened structure, probably indicating the attachment of soft tissue. A similar structure is also present in *P. australis* [58]. Posteriorly, the lateral wing rapidly reduces in size and bears a deep, narrow articular facet for the prefrontal. However, the shape of the naris cannot be reconstructed unambiguously in *Acamptonectes* since the prefrontal, the premaxilla and the anterior part of the lacrimal are missing in both specimens. Unusually, the nasal bears a large foramen between the facet for the prefrontal and the ridge that borders the excavatio internasalis.

The lacrimal (GLAHM 132588) possesses a prominent lateral bony flange on its lateral surface, as is the case in many ichthyosaurs [60]. The anterior edge is not preserved, so it is not possible to know if the lacrimal participated in the ventral margin of the naris or not. The medial surface of the lacrimal is rugose and bears foramina near the suborbital flange.

The postfrontal (SNHM1284-R) exhibits a wide, oblique ridge on its ventral surface, strengthening the supraorbital flange anteriorly. Posteriorly, the postfrontal becomes dorsoventrally compressed. The medial edge of the postfrontal forms a thin, sheet-like ramus that forms the lateral border of the supratemporal fenestra. The anterior and posterior extremities of the bone are not preserved.

A partial right supratemporal (GLAHM 132588; Figure 2C) is preserved. The medial portion of the bone is wide and of complex shape. Anteriorly, it forms a transversely compressed ramus that forms the posteromedial edge of the supratemporal fenestra. This ramus is connected to a sheet-like process that forms the dorsomedial edge of the supratemporal. This process isolates two deep grooves medially: a dorsal and ventral one, which probably buttressed the finger-like posterolateral processes of the parietal. The dorsal surface of the supratemporal bears two rugose bulges that are bordered posteriorly by concave areas. Posteriorly, the supratemporal bears a deep banana-shaped cleft for reception of the cartilaginous part of the paroccipital process. The posteroventral part of the supratemporal is not preserved, so it impossible to known if it extended up to the stapes as it does in *O. icenicus* [43]. The lateral part of the supratemporal is a transversely compressed ramus. Its lateral surface is slightly concave and its ventral edge forms a straight, rugose articulatory surface for the squamosal. The posterior margin of the supratemporal fenestra forms an acute angle, similar to that of *O. natans* [41], but not as acute as in *Athabascasaurus* [61].

The right parietal (SNHM1284-R) is nearly complete, lacking the lateral edge and supratemporal process. Anteriorly, the parietal forms a complex interdigitating articulation with the frontal and/or postfrontal. Anteromedially, the parietal possesses a facet for reception of a posteromedial frontal process, similar to that present in *Athabascasaurus* [61] and *Ophthalmosaurus* [51]. A smooth, saddle-shaped zone that probably represents the posterior margin of the parietal foramen follows this frontal facet posteriorly. The posterolateral part of the parietal possesses prominent grooves and ridges for articulation with the parietal process of the supratemporal. This indicates that the supratemporal buttressed most of the posterior part of the parietal, as it does in *Ophthalmosaurus* [43] and *P. australis* [58], but unlike the condition in *Platypterygius hercynicus* [57] and *Sveltonectes* [34] where the parietal and supratemporal are only in contact laterally. The dorsal surface of the parietal is convex anteriorly and concave posteriorly. There is no parietal ridge. The ventral surface of the parietal is textured by two low, oblique ridges that separate, respectively, three slightly concave areas. The anterior-most ridge is probably the tentorial ridge, but it is markedly less developed than that of *O. icenicus* or *Sveltonectes* [34], [43], [51].

#### Palatal complex

The quadrate (GLAHM 132588, SNHM1284-R; Figure 3H, 3I) is ‘C’-shaped and its condyle is separated from the anterior pterygoid lamella by a long saddle-shaped zone: a strong contrast to *P. hercynicus* [62]. The condyle is short and massive. A deep, smooth groove divides its ventral surface anteriorly. This groove separates a wide, eye-shaped medial section that articulates with the articular from a narrow lateral part that articulates with the surangular. The lateral part forms a prominent bulge posterodorsally. The stapedial facet is oval and its ventral edge is thickened, as it is in numerous ophthalmosaurids [51], [58]. The occipital lamella of the quadrate is damaged yet well expressed in GLAHM 132588. As in *O. icenicus* [43], *Sveltonectes* [34] and *P. australis* [58] this lamella forms an angle of about 120° angle with the pterygoid lamella.

The posterior part of the left pterygoid (GLAHM 132588) is preserved. Unlike in *Sveltonectes*, where a prominent forked process is present [34], the posterior end of the pterygoid of *Acamptonectes* is smooth and slightly concave. The medial surface of the pterygoid closely fits the lateral margin of the basisphenoid, forming two tongue-in-groove structures posterior to the depression receiving the basipterygoid process. The depression is comma-shaped and unusually deep.

The anterior process of the vomer (SNHM1284-R) is laterally compressed. Posteriorly, its dorsal margin is twisted medially for about 30° and forms a prominent vertically projecting dorsal lamella, which is incompletely preserved. The ventral part is incompletely preserved, but the dorsal part of the pterygoid facet is present and similar to that of *O. icenicus* [43].

Two elongated rods are interpreted as the hyoids (SNHM1284-R). As in *P. hercynicus* [62] and *Sveltonectes* [34], one end is rod-like while the other is semi-spatulate.

The stapes (GLAHM 132588, SNHM1284-R; Figure 3F, 3G) is ‘mushroom-shaped’, with a large and approximately square-shaped occipital head and a slender, rod-like shaft (by ichthyosaurian standards). As in *P. australis* [58], the anterior surface of the shaft of SNHM1284-R is straight whereas the posterior surface is bent; the whole shaft is straight in GLAHM 132588. The quadrate process is reduced and thin. The occipital head of three out of the four stapedes lacks the hyoid process sometimes present in *O. icenicus* [43] and *Platypterygius* spp. [57], [58]. The proximal surface of the stapes of GLAHM132588 bears a deep depression anteroventrally. A similar depression is found in *O. icenicus*, but in this taxon this depression is confluent with the outer margin of the occipital surface [43]. The basioccipital–basisphenoid facet is flat and squared. The large opisthotic facet is triangular and faces dorsally.

The posterior surface of the basisphenoid (GLAHM 132588; Figure 3A–C) is obliquely inclined, deeply pitted, and pentagonal. A deep median groove extends along its entire height and forms a cleft on the posterior edge of the ventral surface. The ventral surface is smooth and does not possess posterolateral depressions for the medial lamella of the pterygoid, whereas it does in *Sveltonectes*, *P. australis*, and *Arthropterygius* [34], [55], [58]. The ventral carotid foramen is situated at the center point of the ventral surface of the basisphenoid, unlike in *Arthropterygius* where it opens posteriorly [55]. Posterolaterally, the basisphenoid possesses a large, rugose, rounded depression for articulation with the stapes via a thick cartilage layer. The basipterygoid processes are short, thick, and semicircular in outline, and resemble those of some *O. icenicus* specimens [51] and, to a lesser extend, those of *Brachypterygius* [43], [46], though these are more elongated and wing-shaped. The anterior margin of the basipterygoid process is blade-like and its posteroventral surface forms a wide convexity. The basipterygoid process is separated from the stapedial facet by a paired, twisted groove for the palatine ramus of the facialis (VII) nerve, as it does in *P. australis* [58]. This groove is simple (unpaired) in other ichthyosaurs for which the basisphenoid is adequately known, such as *O. icenicus* and *Brachypterygius* [43], and no groove is preserved in *Sveltonectes* [34]. The most striking feature of the basisphenoid is the shape of the dorsal surface: this does not form a wide plateau as it does in other ichthyosaurs [34], [43], [58], but is developed as a prominent and crest-like dorsal process, providing the posterior surface with a marked pentagonal outline. The posterior part of the parasphenoid is fused to the basisphenoid, with no trace of a suture.

The exoccipital (GLAHM 132588; Figure 3D, 3E) is similar to that of *Brachypterygius* [46], *Mollesaurus* [45], and *O. icenicus* [43] in having a triangular outline in lateral view. *P. australis* [58], *P. hercynicus* (VF, pers. obs.) and *Sveltonectes* [34] differ in having more columnar exoccipitals. The anterior process of the occipital foot is markedly elongated, in contrast to *Sveltonectes* [34]. A small notch on the medial edge of the ventral surface is present on the left exoccipital of GLAHM 132855. This notch matches a small convexity on the lateral margin of the median concavity for the foramen magnum: it seems that the cartilage layer between the basioccipital and exoccipital was minimal, suggesting a mature age [58]. The ventral surface is pitted and slightly convex. Three hypoglossal foramina are present on each side, and the exoccipital also forms the lateral margin of the vagus foramen. The posterior pillar of the exoccipital is twisted but it is not separated from the body of the exoccipital, unlike in *Sveltonectes* [34]. The supraoccipitalsupraoccipital head is large, oval, and markedly deflected anterolaterally, unlike in *P. australis* or *Sveltonectes* [34], [58] where this head has a much smaller ventral extension.

The supraoccipital (GLAHM 132588; Figure 2D–F) is fragmentary. Its dorsal margin forms a boomerang-shaped plateau that articulates with the parietal. The supraoccipital is weakly arched: this contrasts with the condition present in *Platypterygius* spp. [58], [62] and *O. natans* [41], [44] where the supraoccipital is markedly U-shaped. A prominent, obliquely oriented foramen (for a vein according to Kirton [43] or for an endolymphatic duct according to McGowan [60]) is present at the junction between the flattened median ramus and the thickened lateral foot of the supraoccipital. Laterally, a thick layer of spongiose bone surrounds the impression of the otic capsule. As in other ophthalmosaurids [44], [58], this impression is triangular: the elongated channels housing the anterior and posterior semicircular canals form an angle of ca. 170° and the impression for the utriculus is small and rounded.

The basioccipital (GLAHM 132588, SNHM1284-R; Figure 4) condyle is markedly demarcated from the body of the basioccipital by an extensive peripheral ring; in this respect the element is unlike that of most Jurassic and all Cretaceous ophthalmosaurids where an extracondylar area is absent [34], [46], [55], [63]. The surface of this peripheral ring is markedly concave, as it is in *Ophthalmosaurus* [43] and *Mollesaurus* [45]. Its anterior margin is S-shaped in lateral view and forms a prominent rim, which results in a lateral pointed process in dorsal view (Figure 4G), as in *O. icenicus*. In GLAHM 132588, the ventral part of the anterior margin forms a prominent rim as well, whereas it is shallow and flattened (but, admittedly, slightly eroded) in SNHM1284-R. The basioccipital of *Acamptonectes* differs from that of *Ophthalmosaurus* [44] and *Mollesaurus* [45] in lacking a ventral notch. The opisthotic facet is a rugose mound, separated from the extracondylar ring by a narrow, deep vertical notch. The stapedial facet is slightly concave and semi-oval in shape. Its posterior edge is formed by the raised anterior edge of the extracondylar ring. The condyle is rounded, slightly deflected peripherally, and exhibits concentric growth rings, as it does in many specimens of *O. icenicus* [43], [44] and *Brachypterygius extremus* [43]. The posterior notochordal pit is a narrow vertical incision situated above and towards the right of the centre point. The anterior surface of the basioccipital is rugose and strongly pitted. Unusually, the anterior notochordal pit of GLAHM 132588 is set at the center of one of several small but prominent bulges that texture the anterior surface of the basioccipital. This structure is therefore not equivalent to the basioccipital peg. The basisphenoid facets are separated from each other by a shallow notochordal groove in SNHM1284-R, but there is no evidence for a vertical groove between the stapedial and the basisphenoid facets like that present in *P. australis* [58]. The median concavity for the foramen magnum is unique in *Acamptonectes* in being bilobed anteriorly.

The prootic (GLAHM 132588, SNHM1284-R; Figure 5) is rounded and anteroposteriorly compressed, as in most ophthalmosaurids [34]. A thin but prominent process is present on the dorsal half of the medial edge. This process may be homologous to the anteromedial process for attachment of the slips of M. adductor mandibulae externus present but smaller in *P. australis* [58]. The anterior surface of the prootic is densely pitted, suggesting the presence of cartilage. The otic capsule impression is somewhat V-shaped, as it is in some specimens of *Ophthalmosaurus* [43], [44], and thus differs from *Sveltonectes* [34] and *P. australis* [58] where the impression is T-shaped.

As in *O. icenicus* [43], the paroccipital process of the opisthotic (GLAHM 132588, SNHM1284-R; Figure 6) is elongate, slender, and compressed with its long axis directed posterodorsally. In contrast, it is reduced and stout in other ophthalmosaurids such as *Mollesaurus* [45], *P. australis* [58], and some of the Greensand material (VF pers. obs.). The surface of the extremity of the paroccipital process is irregular and concave in GLAHM 132588, suggesting that its extremity was cartilaginous rather than bony, in contrast to SNHM1284-R. The difference between the two lies in the degree of ossification present at the extremity of the paroccipital process. On the lozenge-shaped lateral surface of the opisthotic, the lateral edge of the paroccipital process forms a prominent, oblique ridge for attachment of the M. adductor mandibulae externus, as in *O. icenicus* [43]. A large, oval-shaped basioccipital facet occupies most of the ventral half of the medial surface of the opisthotic. This facet is larger than in other ophthalmosaurids where the opisthotic is adequately described, extending dorsally up to the center point of the otic capsule impression. The vagus foramen is therefore reduced to a small, triangular, and concave area pinched between the basioccipital facet and the medial edge of the impression of the posterior semicircular canal (compare with the wide vagus foramen present in *O. icenicus* for example [43]). Unusually, a basisphenoid facet is present anterior to the deep and narrow groove housing the glossopharyngeal nerve (IX) or the main hyomandibular branch of the facialis nerve (VII) [58]. This groove is also unusual in separating the stapedial facet into a large, elongated, deeply concave lateral part and a minute posteromedial part, which is extremely small because of the large basioccipital facet. The medial part is usually the same size or bigger than the lateral one in other ophthalmosaurids [43], [58]. Anteriorly, the opisthotic forms a massive rectangular foot with a rugose anterior surface. The V-shaped impression of the otic capsule invades the element for a depth of up to 11 mm. As in *Mollesaurus* [45], but in contrast to *P. australis* [58], the ventral part of the impression, housing the posterior ampulla and the sacculus, is not transversely expanded in SNHM1284-R. It is wider and rounder in GLAHM 132588, more closely resembling that of *O. icenicus* [43].

#### Mandible

The dentary (SNHM1284-R; Figure 1) is elongated, straight, and possesses a blunt anterior tip, in contrast to *P. americanus* [64] and *P. australis* [58] where the tip of the rostrum is downturned and beak-like. The fossa dentalis is continuous, deep, and ends anteriorly as a series of aligned foramina (much like the fossa praemaxillaris). Three additional foramina are present on the very tip of the right dentary of SNHM1284-R.

The most anterior part of the splenial (SNHM1284-R, NHMUK R11185; Figure 1) is present 204 mm posterior to the tip of the mandible in SNHM1284-R. Each splenial expands in depth posteriorly to form the mandible's ventral border as well as much as of its medial surface.

In contrast to other ophthalmosaurids [43], [57], [58], the angular (GLAHM 132588, SNHM1284-R; Figure 1) of SNHM1284-R lacks the “3”-shaped dorsal surface. Instead, the dorsal surface is a simple flat groove, bordered by two rounded walls: a small lateral one and a higher medial one. The ventral part of the surangular occupies the lateral part of that groove, the rest being devoted to the Meckelian canal. However, the typical ‘3’-shaped dorsal surface is present in GLAHM 132588, so this feature is possibly variable intraspecifically or ontogenetically.

Posteriorly, the dorsal margin of the surangular (GLAHM 132588, SNHM1284-R; Figure 1) forms a glenoid depression anterior to the contact area with the articular. A low coronoid process is present, but there is no evidence for a M.a.m.e. process like that present in some specimens of *O. icenicus* [43] (VF pers. obs. on GLAHM material) in SNHM1284-R. A very prominent one is present, however, in GLAHM 132588. In contrast to *Sveltonectes* [34], the fossa surangularis is present and ends posteriorly as a large foramen. According to McGowan [60], this probably housed a blood vessel.

The articular (GLAHM 132588) is stouter than in other ophthalmosaurids [34], [51], [55], [58], being nearly as thick as it is long. The articular surface for the quadrate is roughly triangular and markedly flat, whereas it is usually semi-oval [51]. A thick medial ridge connects the anterior and posterior surfaces of the articular, as in *O. natans* [41].

#### Dentition

Only twelve fragmentary teeth (GLAHM 132588, SNHM1284-R; Figure 3J, 3K) are preserved and nine of them (found with NBM1284-R) are just fragmentary roots. These roots are striated basally and some are roughly quadrangular (as is the case in numerous ophthalmosaurids [34]); they are not obviously square-shaped as they are in *Platypterygius* spp. [65] (VF pers. obs.). Some roots exhibit resorption pits, suggesting SNHM1284-R was still growing teeth when it died. Three crowns are preserved and only one is complete. The relative size of the crown is small: its tooth size ratio (TSR, see [34] for the definition of this index) is 1.69 in GLAM 132588, which is slightly higher than that of *O. icenicus* but much smaller than that of *Platypterygius* spp. and *Brachypterygius* (VF, unpublished data). This ratio is, however, based on a single crown and is therefore likely to underestimate the ‘true’ TSR, which should be calculated on the largest crown in the jaws. The crown is slender and sharply pointed, even more so than in *Sveltonectes* [34], but similar to the posterior teeth of *O. natans* [41]. Subtle longitudinal ridges are present only in the basal two-thirds of the crown. This is not due to wear since one of the apices preserved is macroscopically smooth yet sharply pointed. A coarse texture is still present on the entire crown of SNHM1284-R, but this texture is much finer (best seen under microscope under a X25 magnitude) than in *Aegirosaurus* [11] and in some *Platypterygius* specimens [57], [66]. In contrast to other ophthalmosaurids, the base of the crown is slightly bulbous and nearly smooth.

#### Axial skeleton

The atlas-axis (SNHM1284-R; Figure 1) is roughly pentagonal and wide in posterior view: in contrast, in *Arthropterygius* [55] and some specimens of *O. icenicus* [43], [51] it is laterally compressed. It is strongly amphicœlous and approximately 48 mm long. There is no trace of a atlas-axis suture ventrally. The lateral and dorsal surfaces cannot be described accurately since they are only accessible on a cast made in the field during the excavation of the specimen. The anterior surface of the atlas exhibits thick radiating ridges. There is a ventral keel that is separated from the lateral surface by a concave area, as is also present in some *Platypterygius* specimens (VF pers. obs.).

The diapophysis of the centra (GLAHM 132588, SNHM1284-R; Figures 7, 8) remains fused to the neural arch facet in the anterior dorsal region, resulting in a high number of so-called ‘cervical’ centra (nineteen in SNHM1284-R; only a discontinuous series of six cervicals is preserved in GLAHM 132588). This also occurs in such ophthalmosaurids as *P. americanus* [47], ‘*Otschevia*’ [48], and *Sveltonectes* [34]. A wide ridge unites the diapophysis with the neural arch facet of the anterior-most dorsal centra. ‘True’ anterior dorsal centra with diapophyses located in the dorsal half and lacking any connection to the neural arch facets are present as well. As in *Sveltonectes* [34], the dorsal surfaces of the cervical and some dorsal centra are slightly bent, becoming concavo-convex, which presumably served to stiffen the anterior part of the ventral column. However, *Acamptonectes* is unique in exhibiting this bent morphology on the lateral surfaces of some cervical and many dorsal centra, which become S-shaped as well. This is well expressed in GLAHM 132855 and also present - less markedly - in SNHM1284-R. This tight interlocking of the anterior part of the vertebral column, coupled with the robust occiput, seems to accentuate the trend of stiffening the anterior vertebral column seen elsewhere in thunnosaurian ichthyosaurs [21], [67]. The anterior surfaces of the flexural centra are convex peripherally and slightly concave at their centers; their peripheral surfaces are markedly concave and chevron facets are absent, as is also the case in *Sveltonectes* [34], *Arthropterygius* [55], and *Platypterygius* [62], [68]. The vertebral column is regionalized (Figure 8) in a similar way to that of *O. natans* [69], being weak compared to that of *O. icenicus* [69], but more developed than in *Sveltonectes* [34] or *P. platydactylus* [49]. The anterior cervicals are high and then become relatively longer posteriorly. This trend continues to the posterior dorsal centra, where centra become relatively higher, the highest H/L ratio being 3.12 in an anterior caudal centrum of SNHM1284-R. The centra then become relatively longer again and post-flexurals are nearly as long as high (H/L ratio≈1, a condition previously found only in *P. platydactylus* [49]). The diapophyses are prominent, mound-like and pitted. In contrast, the parapophyses are elongated, slightly raised, and bear a central depression. The caudal apophyses are similar to the parapophyses, suggesting that the diapophyses disappear at the beginning of the sacral region, as suggested by McGowan and Motani [2]. The neural arch facets are all very deep and markedly raised with respect to the floor of the neural canal, especially in the cervical and anterior dorsal regions. In the cervical region, the neural arch facets are triangular and become progressively elongated and rectangular in the anterior dorsal region. These facets then progressively shorten in the anterior caudal region.

In contrast to other thunnosaurians [13], the ribs (GLAHM 132588, SNHM1284-R, NHMUK R11185; Figure 7D) are robust with round cross-section, and lack the anterior and posterior grooves that give the rib an 8-shaped cross-section. However, a minute groove is only present on one side of the thoracic ribs (it is not clear which side).

Numerous cervical and thoracic neural arches (GLAHM 132588, SNHM1284-R; Figure 7A) are present, but none is complete. The pre- and postzygapophyses are narrow and unpaired along the whole vertebral column. This is different from the condition present in *P. hercynicus* [62] and *Sveltonectes* [34] where the cervical and anterior dorsal zygapophyses remain paired. However, a subtle ridge separates the prezygapophyses in some cervical or anterior dorsal neural arches. The size of the neural spine varies throughout the ventral column: most dorsal neural spines are slightly lower than the corresponding centra but a few dorsal neural spines in SNHM1284-R are extremely elongated. The largest neural spine reaches 119 mm, which is 1.25 times the height of the largest centra preserved (an anterior caudal one), and >1.3 times the height of all dorsal centra preserved. These high neural spines may be fully ossified versions of the extraneural processes described by McGowan [70] for two specimens of *Stenopterygius*, but no sutures or other signs of intergrowth of two elements are present. The dorsal surfaces of many of the dorsal neural spines are concave and pitted, suggesting the presence of a cartilage cap of unknown extent.

#### Scapular girdle

Both coracoids (SNHM1284-R; Figure 9A, 9D) are preserved and nearly complete. The coracoid is roughly hexagonal with straight, parallel medial and lateral edges, in contrast to the rounded coracoid shape of *Platypterygius* spp. [62], [68]. Both the dorsal and ventral surfaces are slightly saddle-shaped and the medial surface (the intercoracoidal zone) is eye-shaped as it is in *O. icenicus* [51], although it is not as thick as it is in *Sveltonectes* [34] or *P. australis* [71]. The medial surface is unfinished and deeply pitted, indicating the presence of a thick cartilage layer. Anteriorly, the medial margin is strongly deflected laterally and forms the rugose anteromedial edge of a prominent, wide, sheet-like anterior process that is similar to that of *O. icenicus* [50]. This process is separated from the scapula facet by a deep and wide notch, as it is in many *Ophthalmosaurus* specimens [43], [44]. The scapular facet is a small and deeply pitted triangle that is not markedly separated from the large, eye-shaped glenoid contribution; a condition that contrasts with that present in *Sveltonectes*, where those facets are set at a 100° angle [34]. The posterior margin of the coracoid is sheet-like and does not have a notch.

As for the coracoid, the scapula (GLAHM 132588, SNHM1284-R; Figure 9A–C, 9E, 9F) is markedly similar to that of *Ophthalmosaurus* [50], [51]. The shaft is strongly compressed transversely, unlike in *P. hercynicus* where the scapular shaft is thick and rod-like [62]. The ventral part of the scapula is markedly expanded anteroposteriorly, forming a wide, rugose, teardrop-shaped articular surface for articulation with the coracoid and the glenoid. A large, flat acromial process is present anteriorly, as it is in *Ophthalmosaurus* [43], [51] and *P. americanus* [47]. The coracoid facet is triangular and continuous with the larger glenoid contribution, as is the case in *O. icenicus* [51] and but not in *P. australis* [68]. Both the lateral and medial surfaces of the acromial process are slightly concave, Its anterior edge is oblique with respect to the long-axis of the scapula in GLAHM 132588, although not as much as in *Sveltonectes* [34]. A similar range of variation is present in *O. icenicus* (VF pers. obs. on GLAHM material).

#### Forefin

The proximal surface of the humerus (GLAHM 132588, SNHM1284-R, NHMUK R11185; Figure 10A–F) of SNHM1284-R is deeply pitted and roughly trapezoidal in outline, suggesting immaturity. The deltopectoral crest barely extends beyond mid-shaft in SNHM1284-R but extends close to the distal end of the humerus in GLAHM 132588 and NHMUK R11185. The deltopectoral crest of *Acamptonectes* is more prominent than in *Ophthalmosaurus* and *Arthropterygius* [51], [55], [72], but less than in *Sveltonectes* and *Platypterygius* spp. [34], [53], [62], [73]. On the other hand, the trochanter dorsalis is tall and narrow, as in *Sveltonectes* [34] and many species of *Platypterygius* [52], [53]. The posterior edge of the humerus forms an acute trailing blade in SNHM1284-R and NHMUK R11185, but not in GLAHM 132588. Distally, the humerus possesses three facets. The outline and arrangement of these facets is similar to *O. icenicus* [50], [51] and *Arthropterygius* [55]: the facet for the anterior accessory element is small, semi-oval and continuous with the radial facet while the ulnar facet is concave and markedly deflected posteriorly. However, the radial facet is the largest of the three in *O. icenicus* (VF pers. obs. on GLAHM material) and SNHM1284-R, whereas the ulnar facet is the largest in GLAHM 132855. The facet for the anterior accessory elements is smaller than in *Ophthalmosaurus* [43], [51] and *Arthropterygius* [55], reaching only 27 to 31% the size of the radial facet (Table 2), compared to nearly half the size of the radial facet in *Ophthalmosaurus* [41], [43], [51]. The facets are nearly flat and deeply pitted in SNHM1284-R whereas they are well defined, concave, and nearly smooth in the larger GLAHM 132588, suggesting that SNHM1284-R is immature.

The ulna (GLAHM 132588; Figure 10E) is – as is the rest of the appendicular skeleton – much like that of *Ophthalmosaurus* spp. It was so far little noted in the literature that the ulna of *Ophthalmosaurus* spp. is unique amongst neoichthyosaurs: it tapers posteriorly, forming an edgy posterior margin than has a concave profile in dorsal view [41], [43], [51]. Outside *Ophthalmosaurus*, a similar morphology has only been found in the dubious taxa *Yasykovia* and *Platypterygius ochevi* [74], [75]. *Yasykovia* was considered a junior synonym of *Ophthalmosaurus* by both Maisch and Matzke [76] and McGowan and Motani [2]. This may also be the case for *P. ochevi*, but a thorough review of both putative taxa is needed prior to such a reassignment. The ulna described here possesses five articular surfaces. The expanded proximal surface that would have articulated with the humerus is slightly concave and somewhat pitted: in these respects it differs from *Arthropterygius* where the humerus facet of the ulna forms a prominent pyramidal process [55]. The radial facet is straight, trapezoidal in shape, and merges with the facet for the intermedium, which also merges, with the ulnare facet in a smooth transition. The small pisiform facet is triangular and located posterodistally.

Only one phalanx (GLAHM 132588; Figure 10G) is preserved: its large size suggests that it is not a terminal or accessory element. It is oval, as in *Ophthalmosaurus* spp. [41], [51], *Arthropterygius* [55], and some specimens of *Brachypterygius* [43]. It tapers distally and its peripheral edges are irregular and slightly concave.

### Systematic paleontology

cf. *Acamptonectes*

#### Stratigraphy

Upper ‘Neocomian’.

#### Location

Vicinity of Hannover, Germany.

1909 *Ichthyosaurus brunsvicensis* – Broili [77]: 296

1972 *Platypterygius kiprijanoffi* – McGowan [53]: 13

2003 *Platypterygius* – McGowan and Motani [2]: Fig. 38: p. 27; 130

#### Note

Broili [77] described ichthyosaur remains from the ‘Upper Neocomian’ found near Hannover and named it *Ichthyosaurus brunsvicensis*. The material includes an incomplete basicranium (basisphenoid, basioccipital, the right opisthotic, the right stapes, the right quadrate, and both parietals) and an incomplete interclavicle. This material probably belongs to a juvenile individual: the basioccipital is only 55 mm high and the extracondylar area is weakly concave, as in the subadult (SNHM1284-R, see Table 3) and in the smallest specimens (NHMUK and CAMSM material, see below) of *A. densus*.

McGowan [53] considered cranial material to be unreliable for taxonomic purposes and referred the material to the oldest available name given to a ‘Neocomian’ ichthyosaur (*P. kiprijanoffi*) without any detailed comparison of the remains. Notably, this specimen exhibit numerous *Acamptonectes*-like features: a rounded basioccipital with a concave extracondylar band, a long and slender paroccipital process of the opisthotic, a lateral ridge on the opisthotic, a thick lateral foot on the opisthotic, and a stapes with slender shaft and markedly expanded occipital head. However, it differs from *A. densus* in lacking a dorsal crest on the basisphenoid, in having large and laterally expanded basipterygoid process, and there are no indications that it possessed a bilobed median concavity on the basioccipital for the foramen magnum. We maintain ‘*I.*’ *brunsvicensis* as a nomen dubium because of its fragmentary nature and because the original material is not available for study anymore (destroyed during WWII). However, it seems appropriate to refer this specimen to cf. *Acamptonectes*.

### Systematic paleontology

*Acamptonectes* sp.

Figure 11

**Figure 11:**
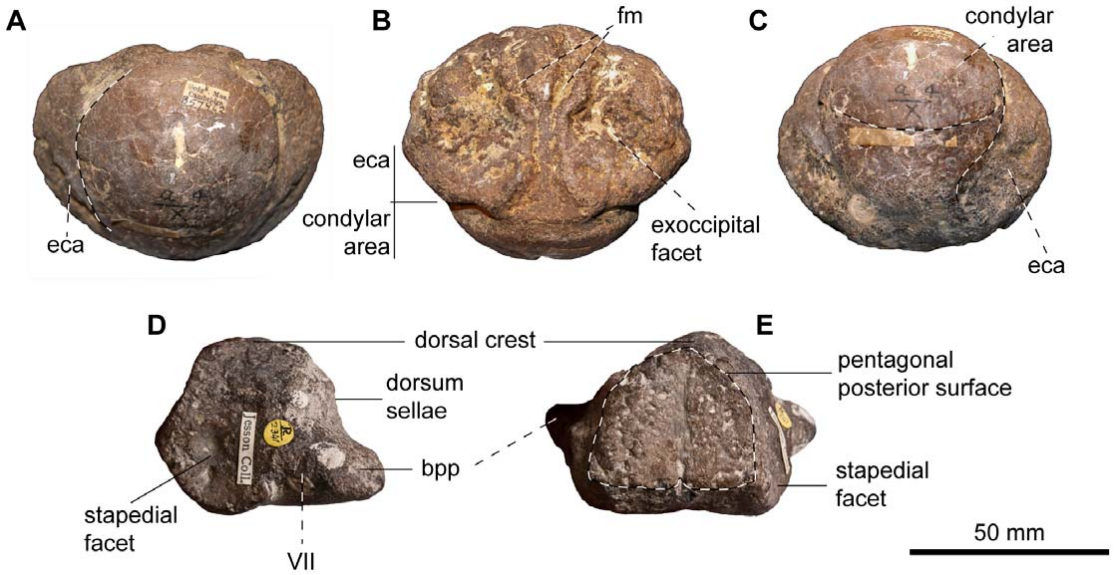
Basioccipital and basisphenoid of *Acamptonectes* sp. from Cambridge Greensand Formation. A–C: basioccipital (CAMSM B57962) in posterior view (A), dorsal view (B), and posteroventral view (C), showing the important extracondylar area (delimited by the broken line). The specimen is the only one of the Cambridge Greensand Formation to exhibit a bilobed concavity for the foramen magnum. D,E; basisphenoid (NHMUK R2341) in lateral (view (D) and posterior view (E), showing the facialis nerve foramen posterior to the basipterygoid process and the pentagonal posterior surface because of the dorsal crest that is characteristic for the genus. Abbreviation: bpp: basipterygoid process; eca: extracondylar area; fm: bilobed concavity for the foramen magnum; VII: foramen for the facialis nerve (VII).

#### Stratigraphy

Cambridge Greensand Formation, lower Cenomanian, but includes upper Albian material reworked from the Gault Formation.

#### Location

Vicinity of Cambridge, Cambridgeshire, England.

#### Referred specimens

CAMSM B57955 (basioccipital), CAMSM B57949 (basioccipital), CAMSM B57942 (basioccipital), CAMSM B57952 (basioccipital), CAMSM B56961 (basioccipital), CAMSM TN1735 partim (basioccipital), CAMSM TN1751 partim (basioccipital), CAMSM TN1753 partim (basioccipital), CAMSM TN1755 partim (basioccipital), GLAHM V.1463 (basioccipital, Newmarket road pits), NHMUK 35301 (basioccipital), CAMSM B58074 (stapes), CAMSM B58075 (stapes), CAMSM B58079 (stapes), CAMSM TN1757 partim (stapes), GLAHM V.1535/1 (stapes), NHMUK R2341 (basisphenoid).

### Description

The basioccipital (Figure 11A–C) is spherical. The condyle is slightly flattened and demarcated from the extracondylar area by a peripheral concave zone, which forms a deep dorsolateral notch in some specimens. The extracondylar area is reduced compared to Early Jurassic ichthyosaurs, but large (36% of the total width, see Table 2) compared to most other ophthalmosaurids [34], [55], [58], [63] and even compared to both specimens of *A. densus*, for which the extracondylar area accounts for 15 to 25% of the total basioccipital width. The extracondylar area is prominent both laterally and ventrally and lacks a ventral notch. The stapedial facet is flattened and oval and the opisthotic facet is similar and does not stand out. The exoccipital facets are oval and rugose. As in *A. densus*, the median concavity for the foramen magnum is paired anteriorly in CAMSM B57942, but appears smooth in the other referred specimens. The anterior surface is oblique, rounded and irregular, and a deep anterior notochordal groove is present in two of the specimens (CAMSM B57955 and CAMSM B57949).

The stapes is nearly identical to that of *A. densus*. The shaft is rod-like and slender and the occipital head is markedly expanded, giving the stapes a ‘mushroom’ shape. A small and hollow triangular area for articulation with the pterygoid is present on the ventral surface of the shaft of some stapedes.

The basisphenoid (NHMUK R2341; Figure 11D, 11E) is small and pentagonal in dorsal view. The anterior edge forms a straight and acute blade, as in *A. densus*. The basipterygoid process is protruding but not as large as in *Platypterygius* [58], *Brachypterygius* [46], or *A. densus*. As in *A. densus*, however, there is deep groove for the facialis nerve (VII) posterior to the basipterygoid process. The basisphenoid is also similar to *A. densus* in having a pentagonal articular surface for the basioccipital, a dorsal crest rather than a plateau, and large stapedial facets whose ventral edge is set below the level of the pterygoid facet.

### Systematic paleontology

cf. *Ophthalmosaurus* Seeley 1874

Figure 12

**Figure 12:**
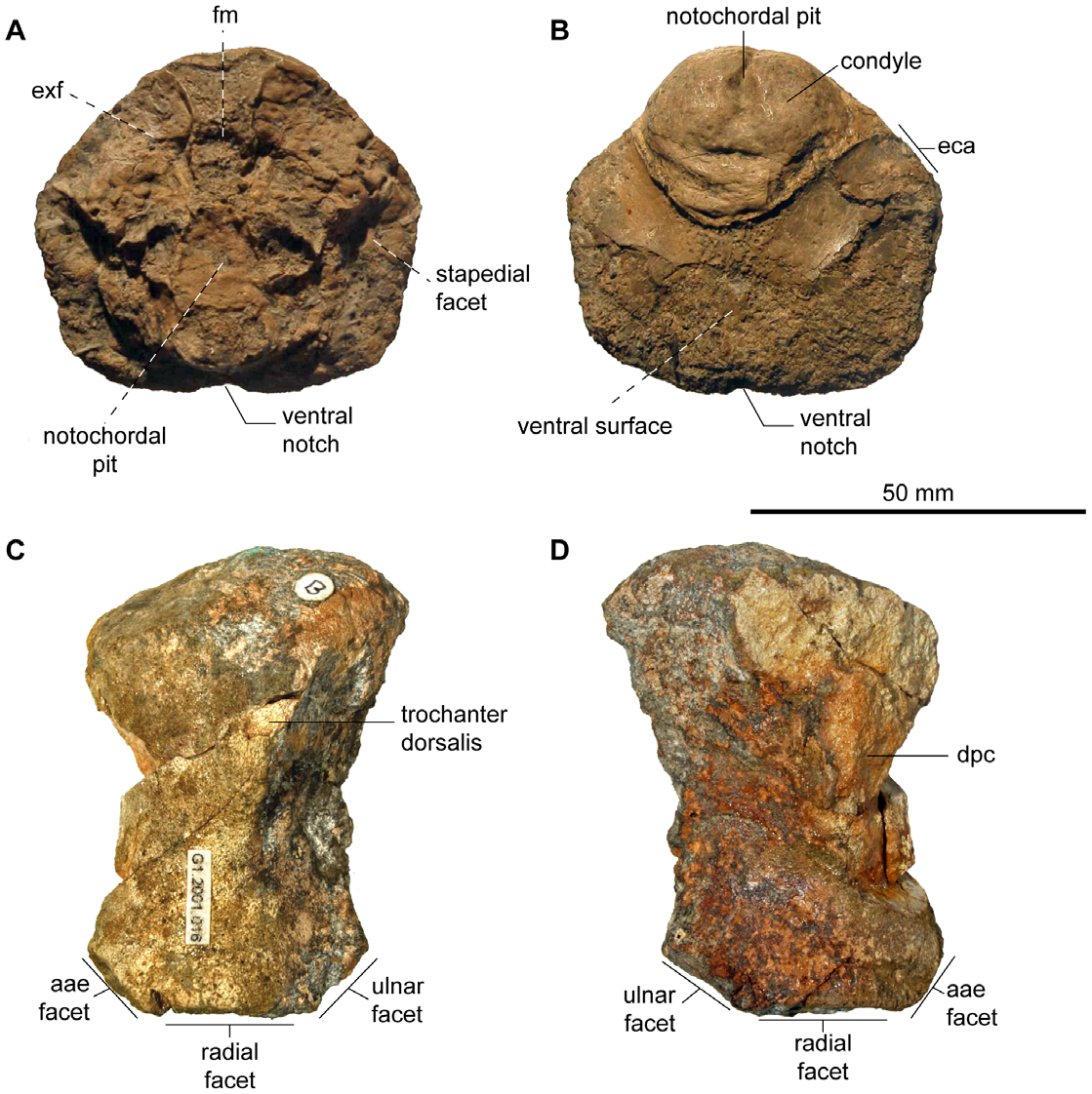
Basioccipital and humerus of cf. *Ophthalmosaurus* from the lower sandstone member of the Spilsby Formation. A,B: basioccipital (LEICT G3.2001.001), in anterodorsal view (A) and posteroventral view (B). The basioccipital is sheared flat, giving the impression of a ‘Liassic-grade’ basioccipital. C,D: left humerus (LEICT G1.2001.016), in dorsal view (C) and ventral view (D). Abbreviation: aae facet: facet for the anterior accessory element; dpc: deltopectoral crest; eca: extracondylar area; exf: exoccipital fact; fm: median concavity for the foramen magnum.

#### Stratigraphy

Lower member of the Spilsby Sandstone Formation, uppermost Tithonian to Berriasian.

#### Location

Nettleton area. See Forrest & Oliver [78].

#### Referred specimen

LEICT G3.2001.001 (basioccipital), LEICT G1.2001.016 (left humerus).

### Description

Forrest and Oliver [78] mentioned the discovery of an ichthyosaur propodial and a ‘Liassic style’ basioccipital in the lower member of the Spilsby Sandstone Formation of the Nettleton area, which was considered Upper Jurassic at the time of publication. However, more recent reassessment consider the lower member of the Spilsby Sandstone Formation as mainly Berriasian [31]. The humerus was found in the basal Spilsby nodule bed (Forrest, pers. comm., 2011) and is probably latest Tithonian in age, whereas the basioccipital was found in the overlying argillaceous sandstone (Forrest, pers. comm., 2011). This sandstone was deposited in the Primitivus Zone [78], and is therefore earliest Berriasian in age [31].

The basioccipital (LEICT G3.2001.001; Figure 12A, 12B) is intensely sheared, as indicated by the anteriorly placed exoccipital facets and median concavity for the foramen magnum. The ventral surface has shifted posteriorly, giving the impression of an extensive extracondylar area, reminiscent of the condition present in Liassic forms. A concave band of bone surrounds the condyle laterally, indicating that it belongs to an ophthalmosaurine ophthalmosaurid (see below for a definition of this new clade name). The median concavity for the foramen magnum is apparently not paired and a subtle ventral notch is present, suggesting that it belongs to *Mollesaurus* or *Ophthalmosaurus*. Given the distant geostratigraphic occurrence of *Mollesaurus* (Bajocian of Neuquén Basin, South America [45]), we refer LEICT G3.2001.001 to cf. *Ophthalmosaurus*.

The Nettleton humerus (LEICT G1.2001.016; Figure 12C, 12D) is consistent with *Ophthalmosaurus*. The deltopectoral crest is low and does not reach midshaft, unlike in *Arthropterygius* which nearly lacks a deltopectoral crest [55] and unlike platypterygiine ophthalmosaurids which have an enlarged, prominent deltopectoral crest (see below). Distally, the humerus possesses three articular facets for the radius, ulna, and an anterior accessory element. These facets are smooth and shallow, unlike in *Arthropterygius* [55]. The facet for the anterior accessory element is relatively larger than in *Acamptonectes*, being approximately half the size of the radial facet (44.9%): in contrast, it is only 27 to 31% the size of the radial facet in *A. densus* (Table 2). A similarly sized anterior accessory facet is found in *Ophthalmosaurus* spp. [41], [43], [51] and *Arthropterygius* [55]. The ulnar facet is deflected posterodistally and the radial facet faces distally, as in non-platypterygiine ophthalmosaurids (*Arthropterygius* and Ophthalmosaurinae).

### Phylogeny

**Figure 13:**
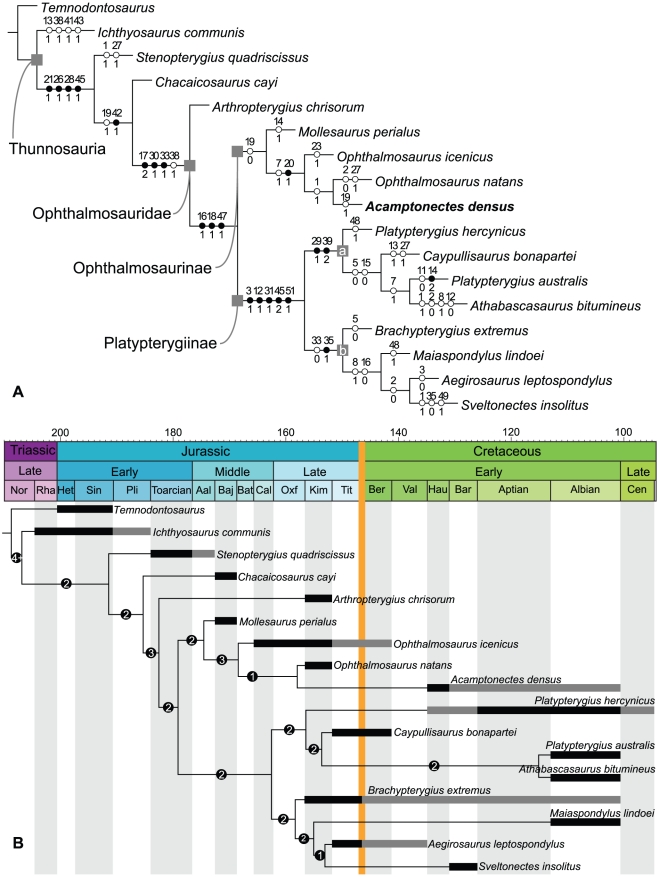
Phylogenetic relationships of Thunnosauria. A: the single most parsimonious tree (length = 101 steps; consistency index = 0.54; retention index = 0.66) in unambiguous optimization. Black circles represent non-homoplastic synapomorphies, open circles represent homoplastic synapomorphies, and numbers above and below circles represent character number and character state, respectively. Ophthalmosauridae rapidly separated in two markedly distinct clades: Ophthalmosaurinae and Platypterygiinae. B: stratigraphy-calibrated phylogeny and bremer support for each branch (white number in plain black circle). The black box represents the stratigraphic range of the species and the gray box expresses the additional range of the corresponding genus. Plurispecific genera (*Ophthalmosaurus* and *Platypterygius*, which may not be monophyletic, see Results–Phylogeny) have their range indicated at the level of one of their species only (*O. icenicus* and *P. hercynicus*, respectively). Many genera and lineages cross the JCB, represented by the vertical orange line. Abbreviations: Aal: Aalenian; Baj: Bajocian; Bar: Barremian; Bat: Bathonian; Ber, Berriasian; Cal: Callovian; Cen: Cenomanian; Hau: Hauterivian; Het: Hettangian; Kim: Kimmeridgian; Nor: Norian; Oxf: Oxfordian; Pli: Pliensbachian; Rha: Rhaetian; Sin: Sinemurian; Tit: Tithonian.

A single most parsimonious tree was recovered from the phylogenetic analysis (Figure 13). It has a length of 101 steps, a consistency index of 0.54 and a retention index of 0.66. The Bremer support (Figure 13B) is significantly higher than in Fischer et al. [34], reaching 2 or 3 at most nodes. However, bootstrap values remain low, and only the clade Ophthalmosauridae is characterized by a bootstrap value higher than 50% (57). All character states discussed below are unambiguous and non-homoplastic, unless stated otherwise.

Our analysis found a similar support for a *Stenopterygius*-ancestry of Ophthalmosauridae as in Fischer et al. [34]. In unambiguous optimization, Ophthalmosauridae is characterized by a reduced extracondylar area (char. 17), a plate-like dorsal trochanter (char. 30), a humerus with a facet for an anterior accessory element (char. 33, reversed in clade ‘B’), and the absence of notching on the paddle elements of the forefin (char. 38, homoplastic). Ophthalmosauridae without *A. chrisorum* are united by the following character states: large basipterygoid processes (char. 16, reversed in the clade *M. lindoei* + *A. leptospondylus* + *S. insolitus*), the absence of a basioccipital peg (char. 18), and the presence of large trochanters on the femur (char. 47).

The main topological novelty of our analysis lies at the base of the ophthalmosaurid lineage, which diverges into two clades: Ophthalmosaurinae, which is quite similar to the basal *Arthropterygius*; and Platypterygiinae, which is markedly derived from the ancestral *Arthropterygius* stock (see below for a phylogenetic definition of these clade names). Both these subfamilies have been informally used in the literature in the past [79], but have never been defined or recovered in cladistics analyses until now. A single feature unites Ophthalmosaurinae: the presence of a ventral notch on the basioccipital (char. 19, homoplastic and reversed in *A. densus*). On the other hand, Platypterygiinae is supported by numerous unambiguous synapomorphies that are easy to spot, even in fragmentary specimens: a quadrangular root section (char. 3), a frontal that participates in the supratemporal fenestra (char. 12, reversed in *A. bitumineus*), the presence of a large, prominent deltopectoral crest (char. 31), absence of the obturator foramen from the ischiopubis (char. 45), and the presence of postaxial accessory digits on the hind fin (char. 51). Two lineages can be recognized within platypterygiine ophthalmosaurids. The unambiguous synapomorphies of clade ‘A’ (*P. hercynicus*, *C. bonapartei*, *P. australis* and *A. bitumineus*) are: a rounded coracoid lacking an anteromedial notch (char. 29) and the presence of two posterior accessory digits in the forefin. Clade ‘B’ (*B. extremus*, *M. lindoei*, *A. leptospondylus* and *S*. *insolitus*) possesses two unambiguous synapomorphies: absence of a facet for an anterior accessory element on humerus (char. 33, homoplastic) and presence of a humerus–intermedium contact (char. 35, reversed in *S. insolitus*).

Our analysis failed to recover monophyletic *Ophthalmosaurus* and *Platypterygius*. The polyphyletic, wastebasket nature of *Platypterygius* has already been noted by other authors [34], [57], [61]. Our consensus tree suggests that *O. natans* should be given a distinct generic moniker since it does not group with the type species of *Ophthalmosaurus*. As is well known, the name *Baptanodon* is already available for it. However, *O. natans* is united with *A. densus* due to a single homoplastic synapomorphy (reduced crown striations, char. 1) and we do not yet consider this sufficient evidence to resurrect use of the name *Baptanodon*. Because of the obvious differences between *Acamptonectes* and the *Ophthalmosaurus* species, we do not consider it preferable to include *Acamptonectes* within an expanded version of *Ophthalmosaurus*. Further study should help clarify the affinities, and hence taxonomy, of these taxa.

### Ophthalmosaurinae Baur 1887

#### Emended diagnosis

Ophthalmosaurids with large extracondylar area of the basioccipital in form of a thick and concave peripheral band; posterodistally deflected ulnar facet of the humerus, large ulna with concave and edgy posterior surface; ischiopubis with obturator foramen.

#### Note

This subfamily was created by Baur [80] under the principle of coordination of the ICZN (chapter 8, article 36).

#### Phylogenetic definition

Branch-based: all taxa closer to *Ophthalmosaurus icenicus* than to *Platypterygius hercynicus*.

### Platypterygiinae Arkhangelsky 2001

#### Emended diagnosis

Ophthalmosaurids with square tooth roots in cross-section; an extremely reduced extracondylar area of the basioccipital; prominent dorsal and ventral trochanters on humerus; ischiopubis lacking an obturator foramen.

#### Note

This subfamily was created by Arkhangelsky [79] but without a formal definition.

#### Phylogenetic definition

Branch-based: all taxa closer to *Platypterygius hercynicus* than *Ophthalmosaurus icenicus*.

### Diversification, extinction, and survival rates

**Figure 14:**
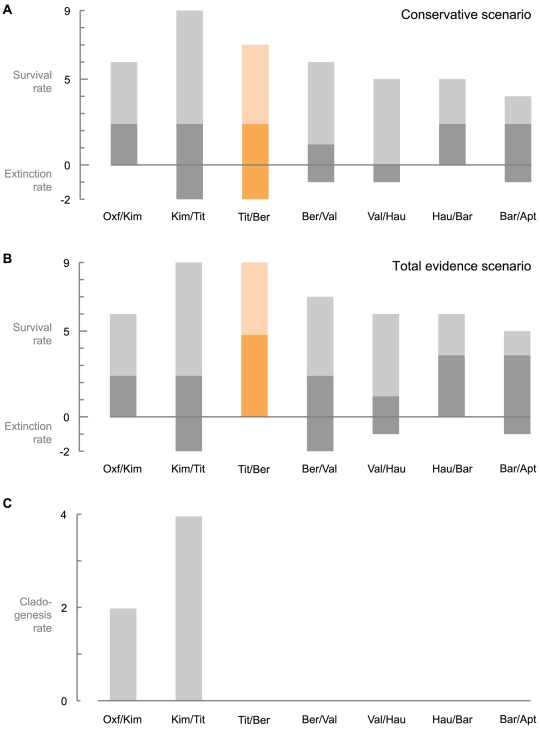
Survival, extinction, and cladogenesis rates of ophthalmosaurids for each boundary of the Oxfordian–Barremian interval. A: survival (positive) and extinction (negative) rates in the conservative scenario, in which post-Jurassic occurrences of both *Ophthalmosaurus* and *Brachypterygius* are ignored. B: survival (positive) and extinction (negative) rates in the total evidence scenario. C: cladogenesis rate. Light color represents phylogenetic lineages and dark color represents genera. Rates for the JCB are colored in orange. The JCB records high survival rates and low to null extinction rates, suggesting that no extinction took place amongst ichthyosaurs at the end of the Jurassic. Abbreviations: Apt: Aptian; Bar: Barremian; Ber, Berriasian; Hau: Hauterivian; Kim: Kimmeridgian; Oxf: Oxfordian; Tit: Tithonian.

Cladogenesis rate (Figure 14C) is higher during the Late Jurassic than during the Early Cretaceous in the interval we considered: it is maximal (4) at the Kimmeridgian–Tithonian boundary and null for the JCB and the whole ‘Neocomian’.

Extinction rates (Figure 14A, 14B) are low for the whole interval we considered, reaching a maximal value of 2 at the Kimmeridgian–Tithonian boundary and at the JCB or Berriasian–Valanginian boundary depending on the scenario considered. Interestingly, the JCB is one of the few boundaries for which the extinction rate is null (under the ‘total evidence’ scenario). No boundary stands out as a particular extinction event, which is confirmed by the elevated survival rates (Figure 14A, 14B). The JCB even records the highest survival rates, both at the generic (4) and phylogenetic (9, as for the Kimmeridgian–Tithonian boundary) levels in the total evidence scenario (Figure 14B), and the second highest (7) in the conservative scenario (Figure 14A).

## Discussion

### The Early Cretaceous ophthalmosaurines of Europe

We found no unambiguous evidence for the presence of *Ophthalmosaurus* in the Cambridge Greensand Formation. However, ophthalmosaurine ophthalmosaurids are definitely present in the ichthyosaur assemblage of this formation. Some of the isolated stapedes are essentially identical to those of *A. densus*, as is one basisphenoid (NHMUK R2341) and one basioccipital (CAMSM B57942). Numerous other basioccipitals are identical to CAMSM B57942, but lack the bilobed median concavity for the foramen magnum. We still refer these specimens to *Acamptonectes* sp. however, since their basioccipital is spherical, lacks a ventrally-expanded extracondylar area, and lacks a ventral notch, in contrast to the condition in other ophthalmosaurine ophthalmosaurids [43], [45]. Generally, the Cambridge Greensand Formation material is of small size and the absence of a paired floor on the foramen magnum could either be an ontogenetic or phyletic feature.

However, our reassessment of the Nettleton material suggests that *Ophthalmosaurus* was still present in England during the early Berriasian. Whereas the combination of features present in this material is diagnostic for *Ophthalmosaurus* (basioccipital with concave extracondylar area, smooth median concavity for the foramen magnum, presence of small deltopectoral crest on the humerus, posteriorly deflected ulnar facet and large facet for anterior accessory element), we refer the Nettleton material to cf. *Ophthalmosaurus* because the specimens concerned are unassociated and originate from different individuals.

Therefore, the peculiar morphologies encountered in the Middle to Late Jurassic genera *Ophthalmosaurus* and *Mollesaurus* are actually diagnostic for a particular and long-living clade of ophthalmosaurids, Ophthalmosaurinae, which persisted at least up to the late Albian, as did their sister-taxon: Platypterygiinae (Figure 13B). Even though the diversity of Early Cretaceous ichthyosaurs has been rapidly growing for the last five years [10], [11], [34], all these new forms belong to Platypterygiinae, which are united by numerous synapomorphies (see Results–Phylogeny). Therefore, the presence of ophthalmosaurine ophthalmosaurids in the Early Cretaceous of Europe represents a major increase in the overall morphological disparity of Early Cretaceous ichthyosaurs. At present, only platypterygiines are known to have crossed the Early–Late Cretaceous boundary, but it remains possible that the poor record of Cenomanian ichthyosaurs obscures the true picture. Moreover, some of the Cambridge Greensand Formation specimens are early Cenomanian in age, so it is conceivable that both ophthalmosaurid clades disappeared during the Cenomanian–Turonian extinction, after a time span of about 80 myr. This indicates the ‘last’ ichthyosaurs were actually taxonomically diverse and morphologically disparate, making their Cenomanian extinction far more severe than previously assumed.

### The effect of the JCB event on ichthyosaurs

Ophthalmosaurids were not affected by the JCB extinction event, as suggested by both the time-calibrated phylogeny and the extinction/survival rates. The extinction rate for the JCB does not surpass the background extinction rate and matches that of the Kimmeridgian–Tithonian boundary in the conservative scenario (Figure 14A). In the ‘total evidence’ scenario (Figure 14B), no ichthyosaur genus goes extinct at the JCB and this boundary records the highest survival rates.

At worst, the JCB event could have triggered a ‘quiescence event’ during which ophthalmosaurids did not go extinct but did not evolve new forms either, as suggested by the null values of the cladogenesis rate during and after the JCB. However, two biases affect these values for the Early Cretaceous: a preservation bias and a taxonomic bias. Indeed, the poor record of earliest Cretaceous ichthyosaurs could explain these low rates, as new Cretaceous forms have simply not yet been discovered. The other bias lies in the poorly understood taxonomy of the genus *Platypterygius*, to which numerous genera have been synonymized (e.g. *Myopterygius*, *Plutoniosaurus*, and *Simbirskiasaurus* [2]). The wastebasket nature of *Platypterygius* was highlighted only recently [57], [81] and the specimens currently included within this genus likely represent a higher generic diversity than currently supposed: in turn, this could reflect higher diversification rates in Early Cretaceous taxa. It is therefore premature to seek explanations for the low cladogenesis rate during the ‘Neocomian’ until a thorough revision of the extensive material referred to *Platypterygius* is conducted.

### Conclusions

*Acamptonectes* nov. gen. is a new ichthyosaur from the Early Cretaceous of Europe that is represented by numerous specimens from the early Hauterivian to the late Albian of England and Germany. It possesses numerous peculiar features previously encountered only in *Ophthalmosaurus* (whose stratigraphic range is now restricted to the Callovian–Berriasian interval) or in the closely related *Mollesaurus*, such as the presence of a concave extracondylar area on the basioccipital, long and slender paroccipital process of the opisthotic, a posterodistally-deflected ulnar facet, a concave and edge-like posterior margin of the ulna, and oval phalanges. Phylogenetic analysis suggests that most of these features actually characterize a clade of ophthalmosaurid ichthyosaurs, Ophthalmosaurinae, which markedly differs from the advanced Platypterygiinae. Both clades rapidly diverged after the appearance of Ophthalmosauridae during the early Middle Jurassic and were still present by the late Albian. In addition to significantly increasing the diversity and disparity of the Cretaceous ichthyosaurs, the presence of ophthalmosaurine ichthyosaurs in the Early Cretaceous of Europe also demonstrates that the JCB extinction event had a negligible effect on ichthyosaurs. Our analysis of cladogenesis, extinction, and survival rates for the Oxfordian–Barremian interval confirms this, and seriously challenges the existence of an extinction event at all for ichthyosaurs at the end of the Jurassic.

## Supporting Information

Text S1 **Description of the characters used in the phylogenetic analysis.**
(DOC)

Text S2 **Nexus file of the character-taxon matrix.**
(TXT)

Table S1 **Character-taxon matrix.**
(DOC)

Figure S1 **Fast (accelerated transformation) and slow (delayed transformation) optimizations.** The characters were optimized on the tree using Winclada [36].
(PDF)
