# Correction: New Ophthalmosaurid Ichthyosaurs from the European Lower Cretaceous Demonstrate Extensive Ichthyosaur Survival across the Jurassic–Cretaceous Boundary

**DOI:** 10.1371/annotation/9731f93a-c28f-4234-8fd9-c587a103b572

**Published:** 2012-01-26

**Authors:** Valentin Fischer, Michael W. Maisch, Darren Naish, Ralf Kosma, Jeff Liston, Ulrich Joger, Fritz J. Krüger, Judith Pardo Pérez, Jessica Tainsh, Robert M. Appleby

Throughout the text, the specimen number GLAHM 132588 of the holotype of the new taxon "Acamptonectes densus" is incorrect. The correct specimen number is GLAHM 132855. 

