# Supplementary material for: New Ophthalmosaurid Ichthyosaurs from the European Lower Cretaceous Demonstrate Extensive Ichthyosaur Survival across the Jurassic–Cretaceous Boundary
Source: PLoS One. 2012 Jan 3;7(1):e29234. doi: 10.1371/journal.pone.0029234 (PMC3250416; doi:10.1371/journal.pone.0029234)
Supplement: Text S1 — Description of the characters used in the phylogenetic analysis. (DOC) [file pone.0029234.s001.doc]

**Text S1. Description of the characters used in the phylogenetic analysis.** Characters are polarized with respect to *Temnodontosaurus* as the outgroup. Most of the characters are taken from Fischer et al.[34]; only the character name and states are indicated, please consult Fischer et al. [34] for the authorship of each of these characters. Modified characters (3, 34) have their modifications underlined. New characters (19, 20, 27, 36) are indicated by an ‘*’.

1. Crown striations: presence of deep longitudinal ridges (0); crown enamel subtly ridged or smooth (1).

2. Base of enamel layer: poorly defined, invisible (0); well defined, precise (1).

3. Shape of the root in cross-section in adults: rounded (0); quadrangular (1).

4. Processus postpalatinis pterygoidei: absent (0); present (1).

5. Maxilla anterior process: extending anteriorly as far as nasal or further anteriorly (0); reduced (1).

6. Descending process of the nasal on the dorsal border of the nares: absent (0); present (1).

7. Processus narialis of the maxilla in lateral view: absent (0); present (1).

8. Processus supranarialis of the premaxilla: present (0); absent (1).

9. Processus narialis of prefrontal: absent (0); present (1).

10. Anterior margin of the jugal: tapering, running between lacrimal and maxilla (0); broad and fan-like, covering large area of maxilla ventrolaterally (1).

11. Sagittal eminence: present (0); absent (1).

12. Processus temporalis of the frontal: absent (0); present (1).

13. Supratemporal-postorbital contact: absent (0); present (1).

14. Squamosal shape: triangular (0); squared (1); squamosal absent (2).

15. Quadratojugal exposure: extensive (0); small, largely covered by squamosal and postorbital (1).

16. Basipterygoid processes: short, giving basisphenoid a square outline in dorsal view (0); markedly expanded laterally, being wing-like, giving basisphenoid a marked pentagonal shape in dorsal view (1).

17. Extracondylar area of basioccipital: wide (0); reduced but still present ventrally and laterally (1); extremely reduced, being nonexistent at least ventrally (2).

18. Basioccipital peg: present (0); absent (1).

19. *Ventral notch in the extracondylar area of the basioccipital: present (0); absent (1).

20. *Shape of the paroccipital process of the opisthotic: short and robust (0); elongated and slender (1).

21. Stapes proximal head: slender, much smaller than opisthotic proximal head (0); massive, as large or larger than opisthotic (1).

22. Angular lateral exposure: much smaller than surangular exposure (0); extensive (1).

23. Posterior dorsal/anterior caudal centra: 3.5 times or less as high as long (0); four times or more as high as long (1).

24. Tail fin centra: strongly laterally compressed (0); as wide as high (1).

25. Neural spines of atlas-axis: completely overlapping, may be fused (0); functionally separate, never fused (1).

26. Chevrons in apical region: present (0); lost (1).

27. *Glenoid contribution of the scapula: extensive, being at least as large as the coracoid facet (0); reduced, being markedly smaller than the coracoid facet (1).

28. Prominent acromion process of scapula: absent (0); present (1).

29. Anteromedial process of coracoid and anterior notch: present (0); absent (1).

30. Plate-like dorsal ridge on humerus: absent (0); present (1).

31. Protruding triangular deltopectoral crest on humerus: absent (0); present (1); present and very large, matching in height the trochanter dorsalis, and bordered by concave areas (2).

32. Humerus distal and proximal ends in dorsal view (thus regardless of the size of the dorsal and ventral processes): distal end wider than proximal end (0); nearly equal or proximal end slightly wider than distal end (1).

33. Humerus anterodistal facet for accessory zeugopodial element anterior to radius: absent (0); present (1).

34. Humerus with posterodistally deflected ulnar facet and distally facing radial facet: no (0); yes (1). The derived state is found in non-platypterygiine ophthalmosaurids. However, ichthyosaurs whose intermedium contacts the humerus (*Aegirosaurus*, *Maiaspondylus*, and *Brachypterygius*) also have a posterodistally facing ulnar facet (and an anterodistally facing radial facet), which is therefore not homologous to that of non-platypterygiine ophthalmosaurids. Thus, a reference to the radial facet has to be included in the character description.

35. Humerus/intermedium contact: absent (0); present (1).

36. *Shape of the posterior surface of the ulna: rounded or straight and nearly as thick as the rest of the element (0); concave and edgy (1).

37. Manual pisiform: absent (0); present (1).

38. Notching of anterior facet of leading edge elements of forefin in adults: present (0); absent (1).

39. Posterior enlargement of forefin: number of postaxial accessory ‘complete’ digits: none (0); one (1), two or more (2).

40. Preaxial accessory digits on forefin: absent (0); present (1).

41. Longipinnate or latipinnate forefin architecture: one (0); two (1) digit(s) directly supported by the intermedium.

42. Zeugo- to autopodial elements flattened and platelike (0); strongly thickened (1).

43. Tightly packed rectangular phalanges: absent, phalanges are mostly rounded (0); present (1).

44. Digital bifurcation: absent (0); frequently occurs in digit IV (1).

45. Ischium-pubis fusion in adults: absent or present only proximally (0); present with an obturator foramen (1); present with no obturator foramen (2).

46. Ischium or ischiopubis shape: plate-like, flattened (0); rodlike(1).

47. Prominent, ridge-like dorsal and ventral processes demarked from the head of the femur and extending up to mid-shaft: absent (0); present (1).

48. Astragalus/femoral contact: absent (0); present (1).

49. Femur anterodistal facet for accessory zeugopodial element anterior to tibia: absent (0); present (1).

50. Tibia peripheral shaft in adults: notched (0); straight (1).

51. Postaxial accessory digit: absent (0); present (1).
