# Supplementary material for: New Ophthalmosaurid Ichthyosaurs from the European Lower Cretaceous Demonstrate Extensive Ichthyosaur Survival across the Jurassic–Cretaceous Boundary
Source: PLoS One. 2012 Jan 3;7(1):e29234. doi: 10.1371/journal.pone.0029234 (PMC3250416; doi:10.1371/journal.pone.0029234)
Supplement: Table S1 — Character-taxon matrix. (DOC) [file pone.0029234.s003.doc]

**Table S1. Character-taxon matrix.** *Temnodontosaurus* as the outgroup. **Abbreviations**: **A**: polymorphism for states 0 and 1; **B**, polymorphism for states 0 and 2.

*Temnodontosaurus* 0000000000 0000000000 0000000000 0000000000 0000000000 0

*I. communis* 0000100A00 001B100000 000000A000 010000A110 1011000001 0

*S. quadriscissus* 1001100000 0000100000 1011?11100 00000010A0 000110?000 0

*O. icenicus* 0101111101 10001A1A01 1110110101 0111011111 1100101001 0

*O. natans* 10?111110? ?0001?1?01 110???110? 011101?1?1 1?00?????? ?

*C. cayi* ?????0???? ??????001? ?????????0 ??0?001000 0100?????? ?

*B. extremus* 011?0100?1 ?????1211? ?1?????101 ?100101111 1100?????? ?

*A. chrisorum* ?????????? ?????0201? 1?11?1??01 011100?1?? 0?????00?? ?

*M. perialus* ??????0?0? ???1111100 1?0??????? ?????????? ?????????? ?

*C. bonapartei* ????00000? ??110????? ?10???1111 1110001121 01102110?1 1

*A. leptospondylus* 000?110111 ?1001????? ?1???????1 ?100101111 1?1021?001 1

*P. australis* 0110001001 0102012110 1101110111 1110001121 0110201011 1

*P. hercynicus* 011?100?01 110A1?2?1? 1??1?10111 1110001121 0110??1101 ?

*M. lindoei* ?11?1?01?1 ?????0???? ??0??????1 ?100101?1? 0?10???10? ?

*A. bitumineus* 10??0011?1 10010?2??? 110?1????? ?????????? ????201??? ?

*S. insolitus* 101?110111 11???0211? 1100?11101 11000??111 1110211011 1

*A. densus* 11???1???? 1????11111 1100?10101 A111011??1 ??0??????? ?
