# Supplementary figures and images for: New Ophthalmosaurid Ichthyosaurs from the European Lower Cretaceous Demonstrate Extensive Ichthyosaur Survival across the Jurassic–Cretaceous Boundary
Source: PLoS One. 2012 Jan 3;7(1):e29234. doi: 10.1371/journal.pone.0029234 (PMC3250416; doi:10.1371/journal.pone.0029234)

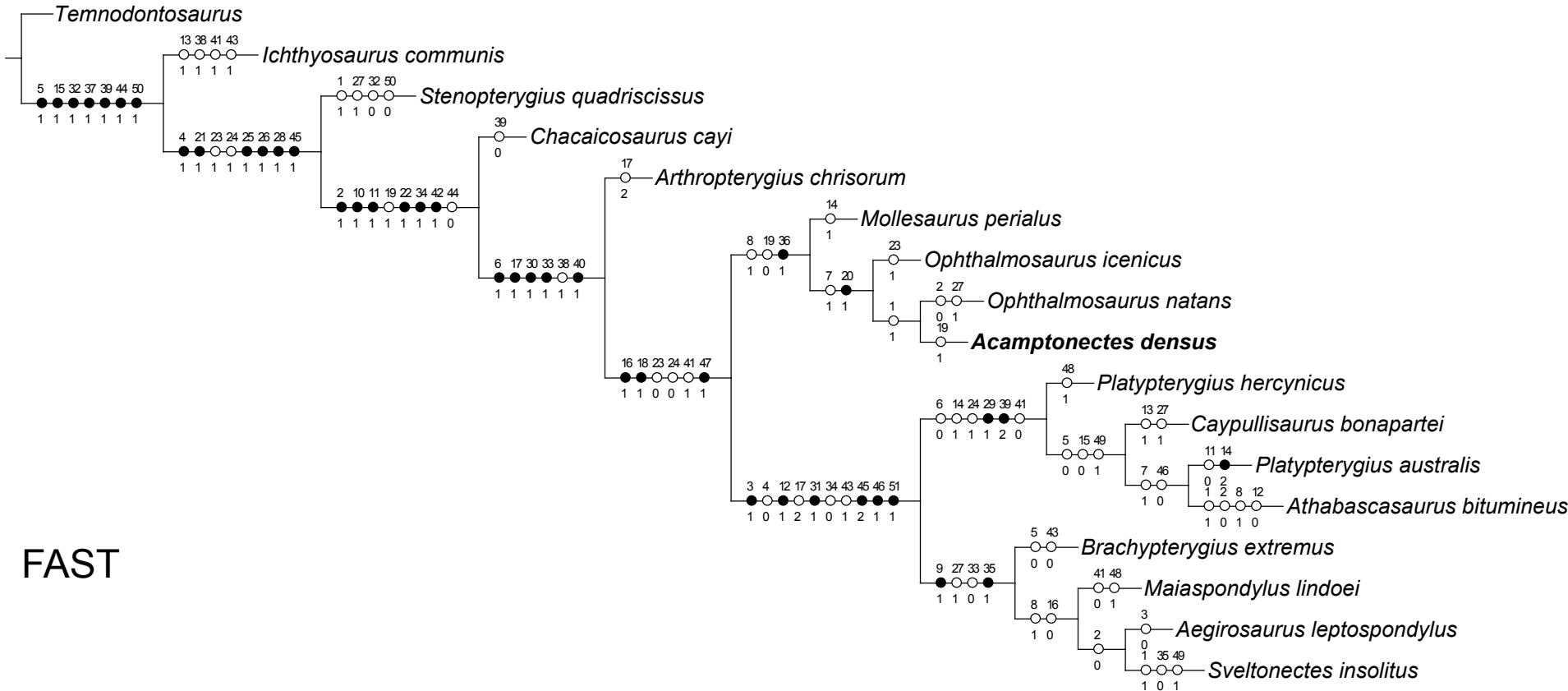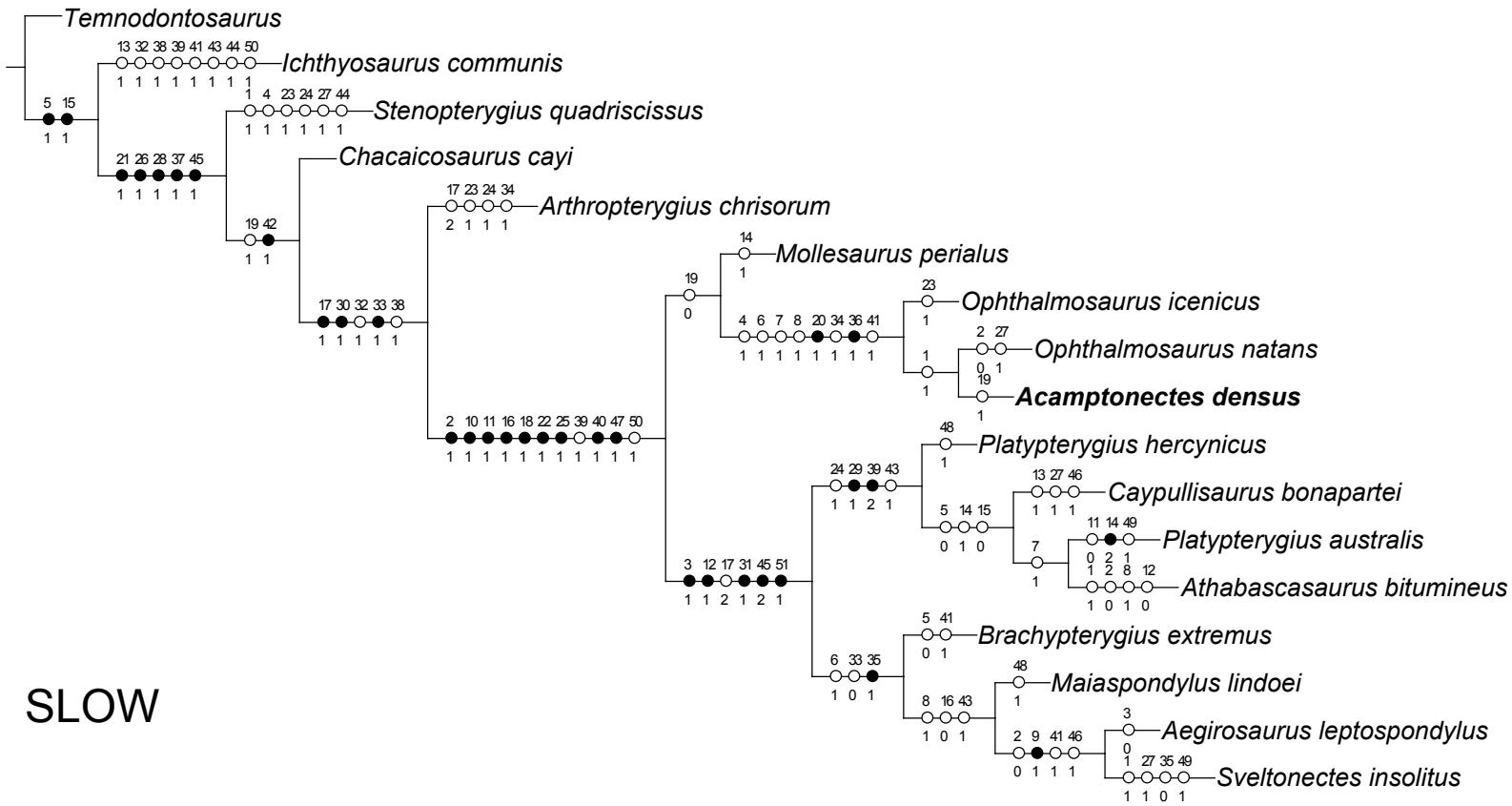

Supplement: Figure S1 — Fast (accelerated transformation) and slow (delayed transformation) optimizations. The characters were optimized on the tree using Winclada [36]. (PDF) [file pone.0029234.s004.pdf]
